# Dietary Energy Level Promotes Rumen Microbial Protein Synthesis by Improving the Energy Productivity of the Ruminal Microbiome

**DOI:** 10.3389/fmicb.2019.00847

**Published:** 2019-04-17

**Authors:** Zhongyan Lu, Zhihui Xu, Zanming Shen, Yuanchun Tian, Hong Shen

**Affiliations:** ^1^The Key Laboratory of Animal Physiology and Biochemistry, College of Veterinary Medicine, Nanjing Agricultural University, Nanjing, China; ^2^College of Life Science, Nanjing Agricultural University, Nanjing, China; ^3^Bioinformatics Center, Nanjing Agricultural University, Nanjing, China; ^4^College of Agriculture, Nanjing Agricultural University, Nanjing, China

**Keywords:** rumen microbiome, energy productivity, substrate-level phosphorylation, electron transport phosphorylation, microbial protein synthesis, dietary modulation

## Abstract

Improving the yield of rumen microbial protein (MCP) has significant importance in the promotion of animal performance and the reduction of protein feed waste. The amount of energy supplied to rumen microorganisms is an important factor affecting the amount of protein nitrogen incorporated into rumen MCP. Substrate-level phosphorylation (SLP) and electron transport phosphorylation (ETP) are two major mechanisms of energy generation within microbial cells. However, the way that energy and protein levels in the diet impact the energy productivity of the ruminal microbiome and, thereafter, rumen MCP yields is not known yet. In present study, we have investigated, by animal experiments and metagenome shotgun sequencing, the effects of energy-rich and protein-rich diets on rumen MCP yields, as well as SLP-coupled and ETP-coupled energy productivity of the ruminal microbiome. We have found that an energy-rich diet induces a significant increase in rumen MCP yield, whereas a protein-rich diet has no significant impacts on it. Based on 10 reconstructed pathways related to the energy metabolism of the ruminal microbiome, we have determined that the energy-rich diet induces significant increases in the total abundance of SLP enzymes coupled to the nicotinamide adenine dinucleotide (NADH) oxidation in the glucose fermentation and F-type ATPase of the electron transporter chain, whereas the protein-rich diet has no significant impact in the abundance of these enzymes. At the species level, the energy-rich diet induces significant increases in the total abundance of 15 ETP-related genera and 40 genera that have SLP-coupled fermentation pathways, whereas the protein-rich diet has no significant impact on the total abundance of these genera. Our results suggest that an increase in dietary energy levels promotes rumen energy productivity and MCP yield by improving levels of ETP and SLP coupled to glucose fermentation in the ruminal microbiome. But, an increase in dietary protein level has no such effects.

## Introduction

Dietary protein for ruminants includes nitrogen (N) occurring in true protein and non-protein. In the rumen, the true protein is degraded into amino acid (AA) and ammonia and then utilized by ruminal microorganisms to synthesize microbial protein (MCP). In the small intestine, more than 80% of rumen MCP is digested, accounting for 50–80% of the total absorbable protein contained there (Tas et al., 1981; Storm et al., 1983). In dairy cows, rumen MCP provides all of the AAs needed for milk protein synthesis (Virtanen, 1966). Because of the high digestibility and good AA composition of MCP, increasing the MCP yield in the rumen is of important significance for the promotion of animal performance. Moreover, increasing the MCP yield is the most effective strategy to reduce the protein feed waste in livestock, since the dietary protein that exceeds the requirement of ruminal microorganisms is degraded to ammonia in the rumen, metabolized to urea in the liver, and lost in the urine.

Previous studies have showed that the sources and amounts of fed carbohydrate are the major factors affecting the energy, in the form of adenosine triphosphate (ATP), available for rumen microbial growth (synthesis of MCP in particular), and that of fed protein affects the production of microbial dry matter (DM) per unit of carbohydrate fermented (Maeng et al., 1976; Hoover and Stokes, 1991). In the dairy cow, 12–13% protein content is needed to maximize the ruminal synthesis of MCP (Satter and Slyter, 1974; Satter and Roffler, 1975). More protein N is incorporated into rumen MCP only if more non-fiber carbohydrate (NFC), known to be the major energy substrate for ruminal microorganisms, are fed to the animals (Schwab et al., 2005). Microbiological studies have revealed that, once the extracellular AA is transported inside microbial cells, the fate of the absorbed AA will depend on the availability of ATPs within the microbial cells. If ATPs are available, AA will be transaminated or used directly for MCP synthesis. However, if ATPs are limited, AA will be deaminated into ammonia and excreted from the cytoplasm (Tamminga, 1979). Accordingly, when dietary protein meets the requirement of ruminal microorganisms, increasing the content of dietary NFC promotes rumen MCP yields via improving the amount of energy supplied to the ruminal microorganisms, whereas increasing the content of dietary protein may have no benefit on rumen MCP yields, since a protein-rich diet might not meet the energy requirement of ruminal microorganisms. So far, much attention has been paid to the ratio of dietary NFC/protein that is optimum for MCP yields (Casper and Schingoethe, 1989; Cameron et al., 1991; Henning et al., 1991). However, the mechanism that the ruminal microbiome applies to producing the energy, and the way that the NFC-rich diet or protein-rich diet impacts the energy productivity of the ruminal microbiota, compared with a basal diet, have not been studied as yet.

It is known that ATP is produced in the microbial metabolism via two major mechanisms: (1) substrate-level phosphorylation (SLP), and (2) electron transport phosphorylation (ETP). In the former, the high-energy phosphate in the substrate molecule is directly donated to ADP to produce ATP. In the latter, electrons that are obtained from energy sources are moved by the electron transport chain (ETC) to reduce oxygen (aerobic respiration) or other oxidized components (anaerobic respiration). Meanwhile, the transmembrane electrochemical potential (ΔμH^+^/ΔμN_a_^+^) generated by the movement of electrons drives the synthesis of ATP in the cell. In the rumen, SLP majorly occurs in the glycolysis and short-chain fatty acids (SCFAs) production. During these processes, the electron generated in the degradation of glucose to pyruvate is transferred from nicotinamide adenine dinucleotide (NADH) to produce lactate, ethanol, butanol and malate (this is referred to as fermentation). ETP (anaerobic respiration) is known to be coupled only with the generation of propionate following succinate pathway and methanogenesis (Tamminga, 1979). Based on the comparison of incubation results using carbohydrate (cellobiose and maltose) and pyruvate as the substrates, ETP was shown to take a minor part in ATP yields, accounting for 20% of the total ATP yields in the carbohydrate fermentation (Demeyer and Van Nevel, 1986). However, recent studies have detected several new ETP-coupled pathways under anaerobic conditions, for example, acetogenesis via the reductive acetyl-CoA pathway (Wood-Ljungdahl pathway) (Schuchmann and Muller, 2014), and butyrate formation via the reduction of crotonyl-CoA (Herrmann et al., 2008). Accordingly, the importance of ETP to the ATP productivity in the ruminal microbiome, as well as the effects of fed NFC/protein on the ETP- and SLP-coupled ATPs productivity, needs to be re-evaluated.

Here, we present the first *in vivo* investigation of the microbial species, pathways and enzymes related to energy metabolism in the rumen, plus a systematic comparison of the energy productivity of the ruminal microbiome in goats receiving a protein-rich or a NFC-rich diet with that of goats receiving a basal diet. We first collected the ruminal microbiota from the goats receiving the three treatments, and compared the rumen MCP yields and fermentation parameters between the groups. Subsequently, by applying metagenome shotgun sequencing and bioinformatics analysis, we reconstructed the pathways related to SLP and the pathways related to ETP in the rumen. Next, we examined the enzymes that catalyze the SLP in these pathways and the enzymes that belong to the ETC and picked out the species whose genome encoded those specific enzyme. Finally, we compared the abundance of the enzymes and the relative abundance of the target species between the groups. By means of the above steps, we aimed to determine the way that the dietary protein or NFC impacted the energy productivity of the ruminal microbiome and, concomitantly to reevaluate the importance of ETP in the ATP productivities of the ruminal microbiome.

## Materials and Methods

### Ethics Statement

This study was approved by the Animal Care and Use Committee of Nanjing Agricultural University, in compliance with the Regulations for the Administration of Affairs Concerning Experimental Animals (No. 588 Document of the State Council of China, 2011).

### Animals

Twenty-four goats (Boer × Yangtze River Delta White, aged 4 months) were randomly allocated into three groups and received a NFC-rich diet (G group, *n* = 8), a protein-rich diet (P group, *n* = 8), or a basal diet (B group, *n* = 8). The ingredients and chemical compositions of the experimental diets are listed in Table 1.

**Table 1:** Ingredient and chemical composition of the experimental diets in the present study.

Item	B^1^	G^1^	P^1^
Ingredient, % of DM			
Guinea grass^2^	90.0	65.0	75.0
Corn flour	0.0	25.0	0.0
Soybean meal	8.0	8.0	23.0
Additive^3^	2.0	2.0	2.0
**Chemical composition**			
DM,%	90.6	89.5	89.7
Crude protein, %DM	9.6	10.0	15.6
Ether extract, %DM	3.0	3.0	2.8
Crude ash, %DM	6.0	5.2	6.1
NFC, %DM^4^	14.1	28.3	16.3
NDF, %DM	67.3	53.6	59.3
ADF, %DM	40.1	29.7	6.0


*DM, dry matter; NFC, non-fiber carbohydrate; NDF, neutral detergent fiber; ADF, acid detergent fiber. ^*1*^The values are means ± SE. ^*2*^*Aneurotepidimu chinense*. ^*3*^The additive was composed of calcium phosphate, limestone, trace mineral salt, and vitamin premix (vitamins A, D, and E). ^*4*^ NFC = 100 – (NDF + CP + ether extract + ash).*

Goats were housed in individual tie-stall barns and had free access to water. To avoid the selection of dietary components and to maintain the desired ratio, a total mixed ratio (TMR) was offered at 0800 and 1700 daily for the 42-day experimental period with the first 14 days being dedicated to adaptation. Feed intake and refusal of individual goats were measured daily during the experiment. The amount of diet offered during days 15–42 was adjusted on a weekly basis to allow at about 10% ort. Feeds were sampled at the beginning and end of the experiment. The DM, ash, crude fat, and crude protein contents of samples were analyzed according to the procedures of AOAC (Cunniff and AOAC International, 1997). The acid detergent fiber (ADF) and neutral detergent fiber (NDF) values of the samples were analyzed according to the procedures of Van Soest et al. (1991).

### Sample Collection, Microbial DNA Extraction, and Metagenome Shotgun Sequencing

Urine samples were collected from 1200 on day 41 to 1200 on day 42 by indwelling catheters that were connected to containers containing 50% HCl, and then, stored at -20°C for the later analysis of rumen MCP. All goats were killed at the local slaughterhouse at 6 h after receiving their morning feed on day 43. Ruminal contents were strained through a 4-layer cheesecloth and immediately subjected to pH measurement. An aliquot (10 mL) of ruminal fluid was immediately stored at -20°C for the extraction of metagenomic DNA. Another aliquot (10 mL) of ruminal fluid was added by 1 mL of 5% HgCl_2_ solution to inactive the microbial proteases, and subsequently, stored at -20°C for the determination of the volatile fatty acids (VFAs) concentration. An aliquot (5 mL) of ruminal fluid was stored at -20°C for the determination of ammonia N.

The metagenomic DNA was extracted from the ruminal fluid by using a Bacterial DNA Kit, following the instruction of the supplier (Omega, Shanghai, China). The DNA concentration was determined in a Nanodrop 1000 (Thermo Fisher Scientific, Wilmington, DE, United States). The integrity of the microbial DNA was evaluated on a 1.0% agarose gel. Metagenomic DNA libraries were constructed by using the TruSeq DNA Sample Prep kit (Illumina, San Diego, CA, United States). Libraries were sequenced via paired-end chemistry (PE150) on an Illumina Hiseq X Ten platform (Illumina, San Diego, CA, United States) at Biomarker Technologies, Beijing, China.

### Rumen MCP and Fermentation Parameters Determination and Analysis

The amount of rumen MCP was calculated from purine derivative excretion in the urine by using the method described by Chen and Gomes (1995) on a spectrophotometer (721, INESA analytical instrument Co., LTD, Shanghai, China). The concentration of ruminal VFAs was determined by using a gas chromatograph (HP6890N, Agilent Technologies, Wilmington, DE, United States) as described by Yang et al. (2012). The ammonia N was determined by using a colorimetric method (Weatherburn, 1967) on a spectrophotometer.

The two-tailed *t*-test was used in the analysis of the rumen MCP amount and rumen fermentation parameters (VFAs concentration, ruminal pH and ammonia N concentration). Differences were considered significant when *p* < 0.05. These analyses were performed by using SPSS software package (SPSS Inc., Chicago, IL, United States).

### Metagenome Shotgun Sequencing Analysis

#### Genome Assembly

Raw reads were first filtered by using FastX v0.0.13 (Gordon and Hannon, unpublished), with a quality cutoff of 20. Reads shorter than 30 bp were discarded from the sample. The reads that were likely to originate from the host and feeds were removed by using DeconSeq v0.4.3 (Schmieder and Edwards, 2011), with the NCBI goat, corn, soybean, and grass genome sequences as references. The remaining high-quality reads of all samples were taken together and then assembled into scaftigs by using IDBA-UD 1.1.1 (Peng et al., 2012) with the standard parameters.

#### Non-redundant Gene Set Construction

Genes were predicted from the scaftigs by using FragGeneScan 1.31 (Rho et al., 2010). Predicted genes from all samples were gathered together to form a large gene set. BLAT v35 (Kent, 2002) was used to construct the non-redundant gene set. Any two genes with more than 95% identity and more than 90% coverage of the shorter gene were picked out, and subsequently, the shorter one was removed from the large gene set.

#### Gene Abundance Calculation

High-quality reads of each sample were mapped to the non-redundant gene set by using Bowtie2 v2.3.4 (Langmead and Salzberg, 2012) with default parameters. MarkDuplicates in the Picard toolkits version 2.0.1 was used to remove the PCR duplicates in the reads, and then, genomeCoverageBed in BEDTools 2.27.0 was employed to calculate the gene coverage. The reads per kilobase of exon model per million mapped reads (RPKM) of the gene, calculated by [gene coverage × 10^6^/(total mapped reads × gene length)], was used to normalize the gene abundance between the treatments.

#### Gene Function Annotation and Pathway Construction

The predicted genes were annotated to the Kyoto Encyclopedia of Genes and Genomes (KEGG) orthology databases via the KEGG Automatic Annotation Server (KAAS) (Moriya et al., 2007), and subsequently mapped to KEGG pathways (Supplementary Table 1). The pathways related to SLP and ETP (for details, see Results) were constructed by using Photoshop CS 8.01 on the background of KEGG pathways. Finally, the enzymes related to SLP, ETC components (details see below), and the enzymes catalyzing the oxidation of NADH during glucose fermentation were picked out according to KEGG Orthology (KO) annotation.

#### Taxonomic Annotation and Relative Abundance Calculation

The high-quality reads of each sample were mapped back to the scaftigs by using Bowtie2. PCR duplicates were removed by means of MarkDuplicates. The coverage of scaftigs in each sample was computed by using genomeCoverageBed and normalized to the relative abundance of 1 000 000. The composition-based rank-flexible classifier Epsilon-NB in FCP v1.0.7 (Parks et al., 2011) was used to assign the taxonomy of the scaftigs, with reference to the in-house reference catalog of 12 607 bacterial and 21 537 archaeal complete genomes downloaded from the NCBI RefSeq database^1^. The relative abundance and taxonomic annotation of each genus are listed in Supplementary Table 2.

#### Metagenome Binning

Metagenome bins were recovered from the scaftigs by using CONCOCT package version 0.4.1 (Alneberg et al., 2014) with 300 as the number of the cluster. The coverage of the received bins was calculated by using ClusterMeanCov.pl in the CONCOCT package. CheckM (Parks et al., 2015) was used to estimate the completeness, contamination, strain heterogeneity and genome size of the received metagenome bins. Unfortunately, only 3/208 bins showed more than 90% completeness and less than 10% contamination, and 22/208 bins showed more than 70% completeness and less than 20% contamination in this study (Supplementary Table 3). Accordingly, scaftigs were used to represent the species in the present study.

#### Gene and Species Abundance Comparison

The abundance of the enzymes and species were both compared by using the Wilcoxon test in *R*, respectively. Differences were considered significant when *p* < 0.05 and | log_2_(G/B)| or | log_2_(P/B)| > 1.

#### Detection of the Major Contributors to SLP Enzymes and the Specific Fermenters in the Ruminal Microbiome

**FIGURE 1:**
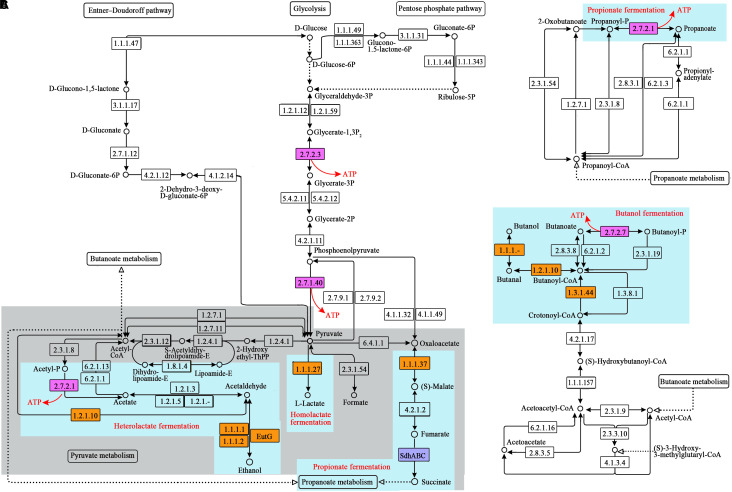
SLP-related pathways reconstructed from the metagenome sequences of the ruminal microbiome. **(A)** Glucose metabolism; **(B)** propanoate metabolism; **(C)** butanoate metabolism. The numbers in the boxes represent the EC numbers of pathway enzymes. The box in pink refers to the SLP enzymes. The box in blue refers to the electron transporter. The box in yellow refers to NADH oxidase during glucose fermentation. Light blue background indicates the key steps of the specific fermentation pathway. ThPP is the abbreviation of meso-5, 10, 15, 20-tetra (4-hydroxylphenyl) porphyrin. KO numbers and gene annotation of the enzymes are shown in Supplementary Table 1.

Based on the gene locations and the taxonomic annotation, the species whose genome encoded the SLP enzymes or the enzymes catalyzing the oxidation of NADH during glucose fermentation were picked out and then summarized at the genus level. In the present study, any genus whose relative abundance was more than 10 in at least one sample was defined as a major contributor to the specific SLP enzyme. The major contributor to all of the NADH oxidases of the specific fermentation pathway, as well as the specific SLP enzyme coupled to the specific fermentation pathway, was considered to be the fermenter to the corresponding product (details see Figure 1).

#### Detection of the Major Contributors to the ETC Components in the Ruminal Microbiome

The ETC is composed of a series of transmembrane ion pumps and F-type ATPase. By checking available publications, we have found the following enzymes that have been reported as ETC components under anaerobic conditions: FADH_2_-NAD oxidoreductase Rnf; membrane-bound hydrogenases Ech and Mbh; methyltransferase Mtr; NADH dehydrogenases Nuo, Ndh, and Hox; cytochrome c reductase Pet and TorC; cytochrome bd oxidase Cyd; sulfate reductase Apr; nitrite reductase NrfA; nitrate reductases Nar and NapA; fumarate reductase Sdh; and cytochrome o oxidase Cyo (Bongaerts et al., 1995; Meuer et al., 1999; Leahy et al., 2010; Fu et al., 2014; De Rosa et al., 2015; Kracke et al., 2015). Except for Ech, Mbh, and Cyo, we detected 13 kinds of enzymes and all subunits of F-type ATPase in the sequences (Table 2). Based on the gene locations and the taxonomic annotation, the species whose genome encoded the ETC components was picked out. Since most enzymes consist of several subunits, only those species whose genome encoded more than half of the subunits of the specific enzyme were used in the subsequent analysis. Finally, the species whose genome encoded the ETC components was summarized at the genus level. The genus whose relative abundance was more than 10 in at least one sample was defined as the major contributor to the specific ETC component in the microbiome.

**Table 2:** Comparisons of the abundances of SLP enzymes, ETC components, and NADH oxidases during glucose fermentation and of the relative abundance of corresponding species between groups.

	PATHWAY	ENZYME	GENE ABUNDANCE					SPECIES RELATIVE ABUNDANCE				
			G^1^	P^1^	B^1^	G/B^2^	P/B^2^	G^1^	P^1^	B^1^	G/B^2^	P/B^2^
SLP enzymes	Glycolysis	EC 2.7.2.3; pgk; phosphoglycerate kinase	1490.58 ± 76.36	1097.76 ± 26.08	1158.74 ± 67.61	0.36	–0.08	1308.5 ± 112.3	1276.4 ± 93.1	1823.2 ± 115.4	–0.48	–0.51
		EC 2.7.1.40; pyk; pyruvate kinase	589.59 ± 25.46	480.02 ± 39.72	436.98 ± 43.85	0.43	0.14	846.5 ± 57.9	751.5 ± 35.9	718.2 ± 37.2	0.24	0.07
	Pyruvate/Propanoate metabolism	EC 2.7.2.1; ackA; acetate kinase	910.36 ± 120.20	625.56 ± 57.83	749.60 ± 63.99	0.28	–0.26	2163.9 ± 185.2	1326.4 ± 111.9	1669.2 ± 137.2	0.37	–0.33
	Butanoate metabolism	EC 2.7.2.7; buk; butyrate kinase	980.68 ± 41.05	410.10 ± 64.37	264.02 ± 130.54	1.89*	0.64	1320.1 ± 99.3	799.1 ± 74.4	518.3 ± 46.7	1.35*	0.62
	Nitrogen metabolism	EC 2.7.2.2; arcC; carbamate kinase	202.21 ± 10.26	174.72 ± 46.94	152.81 ± 28.83	0.40	0.19	479.8 ± 24.5	448.3 ± 32.0	431.3 ± 32.1	0.15	0.06
NADH oxidases in glucose fermentation	Heterolactic fermentation	EC 1.2.1.10; adhE; acetaldehyde dehydrogenase	663.37 ± 20.90	208.92 ± 20.27	227.83 ± 10.88	1.54*	–0.13	1.7 ± 0.5	0.3 ± 0.2	1.1 ± 0.1	0.68	–1.79*
		EC 1.1.1.1; adhP; alcohol dehydrogenase	14.74 ± 2.87	4.75 ± 1.14	4.29 ± 1.33	1.78*	0.15	436.7 ± 22.7	460.2 ± 34.1	632.7 ± 68.3	–0.53	–0.46
		EC 1.1.1.1; adh; alcohol dehydrogenase	8.15 ± 1.77	5.66 ± 1.65	3.87 ± 1.31	1.07*	0.55	32.8 ± 4.5	28.2 ± 3.1	9.9 ± 1.1	1.73*	1.51*
		EC 1.1.1.1; adhC; alcohol dehydrogenase	16.55 ± 6.07	2.01 ± 1.40	6.49 ± 1.48	1.35*	–1.69*	8.4 ± 1.1	4.5 ± 0.6	8.4 ± 0.4	–0.01	–0.89
		EC 1.1.1.1; yiaY; alcohol dehydrogenase	121.40 ± 10.44	32.83 ± 5.67	38.95 ± 7.32	1.64*	–0.25	16.8 ± 1.2	7.0 ± 1.1	11.0 ± 0.5	0.61	–0.67
		EC 1.1.1.2; adh; alcohol dehydrogenase (NADP+)	79.88 ± 11.90	81.79 ± 17.51	78.46 ± 18.96	0.03	0.06	53.6 ± 4.2	94.3 ± 9.7	79.0 ± 4.1	–0.56	0.26
		EutG; alcohol dehydrogenase	40.05 ± 5.12	13.59 ± 3.35	12.94 ± 3.21	1.63*	0.07	116.1 ± 6.8	164.8 ± 9.5	217.6 ± 5.8	–0.91	–0.40
	Homolactic fermentation	EC 1.1.1.27; ldh; L-lactate dehydrogenase	118.76 ± 33.54	125.58 ± 27.77	86.46 ± 15.35	0.46	0.54	125.6 ± 5.2	128.1 ± 5.9	140.9 ± 7.7	–0.17	–0.14
	Butanol fermentation	EC 1.3.1.44; ter; *trans*-2-enoyl-CoA reductase (NAD+)	0.62 ± 0.01	0.62 ± 0.01	0.97 ± 0.00	–0.64	–0.64	725.3 ± 54.7	962.8 ± 68.5	703.4 ± 55.6	0.04	0.45
		EC 1.1.1.-; bdhAB;butanol dehydrogenase	833.56 ± 48.40	414.17 ± 20.41	451.46 ± 20.64	0.88	–0.12	436.7 ± 22.7	460.2 ± 34.1	632.7 ± 68.3	–0.53	–0.46
		EC 1.2.1.10; adhE; acetaldehyde dehydrogenase	663.37 ± 20.90	208.92 ± 20.27	227.83 ± 10.88	1.54*	–0.13	1.7 ± 0.5	0.3 ± 0.2	1.1 ± 0.1	0.68	–1.79
	Propionate fermentation	EC 1.1.1.37; mdh; malate dehydrogenase	612.69 ± 54.98	415.07 ± 96.21	383.46 ± 55.67	0.68	0.11	62.9 ± 3.8	33.6 ± 3.6	34.5 ± 3.2	0.86	–0.04
ETC components	ATPase	ATPase; F-type H+-transporting ATPase	768.75 ± 76.10	382.62 ± 57.24	353.58 ± 41.08	1.12*	0.11	1926.0 ± 197.8	1629.1 ± 187.1	1601.6 ± 192.2	0.27	0.02
	FADH_2_-NAD oxidoreductase^3^	Rnf; electron transport complex protein Rnf	910.04 ± 66.10	417.25 ± 67.79	443.12 ± 51.49	1.04*	–0.09	2295.6 ± 191.3	1574.6 ± 149.0	1424.0 ± 120.8	0.69	0.15
	Methanogenesis	Mtr; tetrahydromethanopterin S-methyltransferase	2.20 ± 0.74	1.96 ± 0.29	2.09 ± 0.46	0.07	–0.09	7.0 ± 0.8	4.3 ± 0.5	6.8 ± 1.3	0.04	–0.65
	NADH dehydrogenase^3^	Nuo; NADH-quinone oxidoreductase	230.44 ± 22.47	120.09 ± 20.18	113.48 ± 11.59	1.02*	0.08	2253.7 ± 176.9	1378.7 ± 155.5	1014.8 ± 73.2	1.15*	0.44
		Ndh; NAD(P)H-quinone oxidoreductase	13.78 ± 3.49	12.97 ± 3.49	10.58 ± 2.85	0.38	0.29	20.0 ± 2.1	8.2 ± 1.2	12.2 ± 0.6	0.71	–0.57
		Hox; bidirectional [NiFe] hydrogenase diaphorase	13.74 ± 3.20	7.52 ± 1.14	10.89 ± 2.14	0.34	–0.53	5.2 ± 0.7	2.1 ± 0.3	5.8 ± 0.6	–0.16	–1.45*
	Cytochrome complex^3^	Pet; ubiquinol-cytochrome c reductase	0.71 ± 0.21	0.89 ± 0.14	0.63 ± 0.11	0.16	0.51	0	0	0	0	0
		TorC; trimethylamine-N-oxide reductase	0.47 ± 0.03	0.83 ± 0.04	0.65 ± 0.07	–0.45	0.36	5.4 ± 1.1	2.8 ± 0.6	3.8 ± 0.4	0.50	–0.47
		Cyd; cytochrome bd ubiquinol oxidase	412.15 ± 47.13	539.51 ± 33.78	366.26 ± 50.13	0.17	0.56	2035.6 ± 212.7	1044.0 ± 116.0	911.6 ± 68.2	1.16*	0.2
	Sulfur dissimilation	Apr; adenylylsulfate reductase	0.64 ± 0.06	0.59 ± 0.05	0.78 ± 0.31	–0.28	–0.39	2.8 ± 0.4	19.9 ± 2.1	24.6 ± 2.0	–3.13*	–0.31
	Nitrogen dissimilation	NrfA; nitrite reductase (cytochrome c-552)	403.53 ± 65.93	202.95 ± 29.52	176.38 ± 24.09	1.19*	0.20	541.3 ± 45.7	337.5 ± 40.5	259.7 ± 22.2	1.06*	0.38
		Nar; nitrate reductase / nitrite oxidoreductase	0.55 ± 0.09	1.60 ± 0.15	1.72 ± 0.22	–1.63*	–0.10	0.2 ± 0.1	8.5 ± 0.9	3.1 ± 0.2	–3.90*	1.46*
		NapA; periplasmic nitrate reductase NapA	0.82 ± 0.03	1.89 ± 0.12	0.83 ± 0.31	–0.02	1.20*	0.34 ± 0.13	4.62 ± 1.08	1.56 ± 0.28	–3.76*	1.57*
	Fumarate respiration	Sdh; succinate dehydrogenase	1210.06 ± 130.02	578.79 ± 32.16	500.64 ± 64.78	1.27*	0.21	1422.9 ± 114.3	1179.1 ± 95.8	976.6 ± 79.6	0.54	0.27


*^1^ mean value ± standard error; the unit of gene results is RPKM; the relative abundance of species results was normalized to 1,000,000 for each sample. ^*2*^ the numbers were calculated from log_*2*_(G/B) or log_*2*_(P/B). ^*3*^ the electron transporters were reported in many kinds of ETCs. ^∗^*p* < 0.05 and | value| > 1 in the wilcox test between groups.*

#### Result Visualization

The comparisons of the relative abundances of the species were visualized by using R program ggplot2.

### Data Submission

The metagenome sequences are submitted to the NCBI under BioProject PRJNA492173.

## Results

### Comparison of Rumen Fermentation Parameters Between Groups

As shown in Table 3, rumen MCP, ammonia N, and total VFAs were significantly increased, whereas ruminal pH was significantly decreased when the diet shifted from diet B to G. Similarly, rumen pH was significantly decreased, whereas ammonia N and total VFAs were significantly increased when the diet shifted from B to P.

**Table 3:** Comparisons of rumen pH, ammonia N, MCP, and VFAs concentrations between groups.

Item	B^1^	G^1^	P^1^
pH	6.74 ± 0.04	6.38 ± 0.05a	6.47 ± 0.03b
Ammonia N, mM	3.04 ± 0.17	8.73 ± 0.73a	12.51 ± 0.87b
MCP, g/d	8.47 ± 0.86	11.49 ± 1.22a	9.31 ± 1.07
Total VFAs, mM^2^	53.01 ± 2.00	78.67 ± 2.93a	67.66 ± 3.57b
VFA, molar proportions			
Acetate	67.7 ± 2.2	58.8 ± 2.7 a	70.6 ± 2.9b
Propionate	21.1 ± 1.4	29.3 ± 2.4 a	18.8 ± 2.6
Butyrate	11.2 ± 1.5	11.9 ± 1.4a	10.6 ± 1.4


*^*1*^Values are mean ± standard error (SE). ^*2*^Total VFA = Acetate + Propionate + Butyrate. ^*a*^ *p* < 0.05 in the two-tailed *T*-test of B group and G group. ^*b*^ *p* < 0.05 in the two-tailed *T*-test of B group and P group.*

With regard to the concentrations of individual VFAs, all of them were significantly increased when the diet shifted from B to G. On the other hand, acetate was significantly increased, whereas others VFAs revealed no significant changes when the diet shifted from B to P.

### Pathways Related to Energy Metabolism in the Rumen Microbiome

**FIGURE 2:**
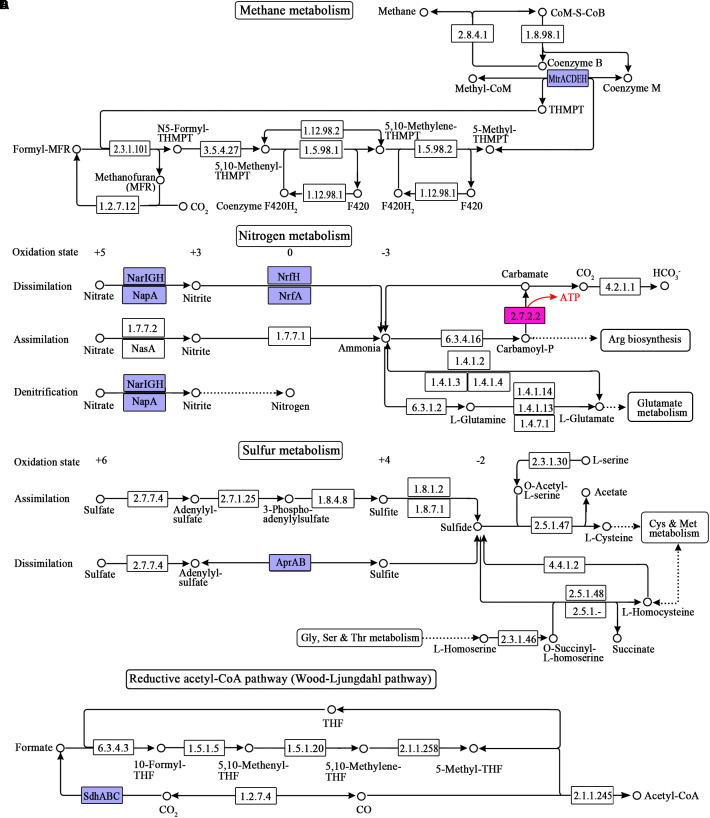
ETP-related pathways reconstructed from the metagenome sequences of the ruminal microbiome. **(A)** Methane metabolism; **(B)** nitrogen metabolism; **(C)** sulfur metabolism; **(D)** reductive acetyl-CoA pathway (Wood-Ljungdahl pathway). The numbers in the boxes represent the EC numbers of pathway enzymes. The box in pink refers to the SLP enzymes. The box in blue refers to the electron transporter. THF is the abbreviation of tetrahydrofuran. THMPT is the abbreviation of tetrahydromethanopterin. KO numbers and gene annotation of the enzymes are shown in Supplementary Table 1.

In the rumen, monosaccharides are degraded into pyruvate through glycolysis, the pentose phosphate pathway, and the Entner-Doudoroff (ED) pathway. Pyruvate is subsequently fermented into formate, acetate, propionate, butyrate, lactate, and ethanol through pyruvate metabolism, propanoate metabolism, and butanoate metabolism. Accordingly, we reconstructed these pathways related to SLP-coupled energy metabolism (Figure 1). To date, ETP has been reported in the dissimilatory reduction of inorganic molecules (such as N and S), fumarate reduction, metals reduction, methanogenesis via the reduction of carbon dioxide (CO_2_), acetogenesis via the reductive acetyl-CoA pathway, and butyrate formation via the reduction of crotonyl-CoA. Therefore, we reconstructed the following pathways related to ETP-coupled energy metabolism: methane metabolism, nitrogen metabolism, sulfur metabolism, and the reductive acetyl-CoA pathway, based on the genes detected in the sequences (Figure 2). Here, we have named the pathways according to the pathway names in the KEGG dataset.

### Comparison of Abundance of SLP Enzymes and Their Major Contributors Between the Groups

From the above pathways, we detected 5 kinds of SLP enzymes (Figure 1, 2 and Table 2), and the abundances of these enzymes were high in the gene pools of the ruminal microbiome (153–1491). The total abundance of SLP enzymes was 2762, 4173, and 2788 in B, G, and P group, respectively. According to the wilcoxon test, it showed the significant increase (*p* < 0.05) when the diet shifted from B to G and no significant change when the diet shifted from B to P. For the individual SLP enzymes, only the abundance of butyrate kinase (buk; EC 2.7.2.7), which catalyzes the reaction of butanoyl-P and ADP to form butanoate and ATP, was significantly increased when the diet shifted from B to G (*p* < 0.05).

Within the ruminal microbiome, 21 of the 68 detected archaeal genera and 258 of the 549 detected bacterial genera included the species whose genome encoded the SLP enzymes (in addition to unclassified genera). However, the changes in the relative abundance of these microorganisms did not correspond to the changes in the total abundance of the SLP enzymes (Table 2), which showed a slight increase when the diet shifted from B to G (0.52–0.61%), and a significant decrease when the diet shifted from B to P (0.52–0.46%, *p* < 0.05). Although at least 8 G of high-quality data was received for each sample, around 90% metagenome bins had less than 70% completeness in the threshold of less than 20% contamination in the present study, possibly because the diversity and abundance of the ruminal microbiome are greater than we are aware. The diet-induced changes in the relative abundance of the major contributors to the individual SLP enzymes are shown in Supplementary Figure 1.

### Comparison of Abundance of Enzymes Catalyzing the Oxidation of NADH in the Glucose Fermentation and the Specific Fermenters Between the Groups

Under anaerobic conditions, NADH produced by glycolysis is oxidized back to NAD^+^ by using pyruvate or one of its derivatives as an electron acceptor. In the present study, we analyzed the enzymes that catalyzed the oxidation of NADH during glucose fermentation between the groups. As a result, 11 enzymes belonging to 4 types of fermentation (heterolactic fermentation, homolactic fermentation, butanol fermentation, and propionate fermentation) were detected (Table 4). Among them, the acetaldehyde dehydrogenase (adhE; EC 1.2.1.10) was the common enzyme used in the heterolactic fermentation and butanol fermentation. In a comparison of their abundances, five alcohol dehydrogenases (EC 1.1.1.1 and EutG) and adhE were significantly increased when the diet shifted from B to G. On the other hand, the abundance of one alcohol dehydrogenase (EC 1.1.1.1) was significantly decreased when the diet shifted from B to P (Table 2).

**Table 4:** Comparisons of the relative abundance of specific fermenters between groups.

Product	Fermenter	B^1^	G^1^	P^1^	G/B^2^	P/B^2^
Butanol	Prevotella	295.2 ± 27.1	859.3 ± 68.9	439.3 ± 44.8	1.54*	0.57
	Syntrophus	10.6 ± 1.1	52.7 ± 2.1	0	2.32*	
	Granulicella	33.3 ± 0.9	44.2 ± 5.4	0	0.41	
	Clostridium	51.5 ± 5.0	38.2 ± 2.3	27.3 ± 1.1	–0.43	–0.92
	Paenibacillus	26.2 ± 2.7	27.7 ± 3.4	26.2 ± 2.0	0.08	0.00
	Selenomonas	8.6 ± 1.0	19.9 ± 1.9	0	1.22*	
	Eggerthella	16.4 ± 1.8	17.8 ± 1.5	30.0 ± 2.2	0.12	0.87
	Slackia	1.7 ± 0.1	15.8 ± 2.5	0	3.23*	
	Ethanoligenens	0.9 ± 0.1	11.7 ± 1.1	0	3.74*	
	Geobacter	26.3 ± 2.6	11.0 ± 0.8	10.5 ± 0.9	–1.26*	–1.32*
	Bacteroides	12.0 ± 0.8	0	47.0 ± 3.8		1.97*
	Syntrophobacter	1.4 ± 0.2	0	15.2 ± 1.6		3.46*
	Bacillus	3.5 ± 0.4	0	14.0 ± 1.3		1.99*
	Staphylococcus	26.6 ± 3.3	0	10.7 ± 0.4		–1.32*
	Butyrivibrio	0.7 ± 0.1	0	10.2 ± 1.3		3.78*
Heterolactate	Clostridium	75.9 ± 6.7	82.8 ± 4.5	78.4 ± 5.5	0.13	0.05
	Paenibacillus	18.6 ± 1.2	60.9 ± 2.5	0	1.71*	
	Lactobacillus	1.9 ± 0.1	26.3 ± 1.4	0	3.81*	
	Oscillibacter	2.7 ± 0.2	22.8 ± 1.4	0	3.08*	
	Sinorhizobium	3.7 ± 0.3	20.0 ± 3.3	0	2.44*	
	Syntrophus	20.0 ± 2.1	18.3 ± 0.4	0	–0.13	
	Desulfovibrio	3.4 ± 0.6	17.1 ± 1.5	0	2.33*	
	Marinobacter	1.4 ± 0.1	14.9 ± 2.0	0	3.36*	
	Gluconobacter	2.1 ± 0.3	13.4 ± 1.2	0	2.69*	
	Prevotella	51.7 ± 3.1	11.3 ± 0.8	57.4 ± 6.7	–2.20*	0.15
	Syntrophobacter	10.8 ± 1.0	10.3 ± 0.5	0	–0.08	
	Bacillus	3.0 ± 0.1	0	44.9 ± 3.8		3.89*
	Geobacter	28.8 ± 3.1	0	24.2 ± 2.5		–0.25
	Treponema	3.3 ± 0.4	0	20.5 ± 2.6		2.63*
	Bacteroides	7.8 ± 1.1	0	14.0 ± 1.3		0.83
	Sphaerochaeta	3.8 ± 0.3	0	13.2 ± 1.3		1.81*
	Ethanoligenens	5.1 ± 0.4	0	12.0 ± 0.8		1.23*
Homolactate	Paenibacillus	7.2 ± 0.7	28.0 ± 1.4	0	1.95*	
	Ruminococcus	2.6 ± 0.6	22.2 ± 0.2	0	3.07*	
	Clostridium	8.8 ± 0.7	0	54.4 ± 2.7		2.63*
Propionate	Granulicella	30.1 ± 2.2	177.3 ± 14.7	12.5 ± 1.1	2.56*	–1.27*
	Prevotella	166.0 ± 13.7	119.8 ± 4.9	341.3 ± 31.5	–0.47	1.04*
	Desulfomicrobium	28.8 ± 1.9	89.8 ± 8.6	12.9 ± 1.4	1.64*	–1.16*
	Acidovorax	6.4 ± 0.6	52.4 ± 5.2	0	3.04*	
	Bacteroides	20.2 ± 1.8	39.6 ± 1.9	30.2 ± 2.9	0.97	0.58
	Geobacter	46.4 ± 3.0	25.2 ± 3.0	57.7 ± 5.5	–0.88	0.31
	Clostridium	15.1 ± 1.1	0	28.6 ± 2.7		0.92


*^1^ mean value ± standard error; the relative abundance was normalized to 1,000,000 for the sample. ^*2*^ the numbers were calculated from log_*2*_(G/B) or log_*2*_(P/B). ^∗^*p* < 0.05 and |value| > 1 in the wilcox test between groups.*

Within the ruminal microbiome, other than the unclassified genera, we detected 10 butanol fermenters, 11 heterolactic fermenters, 2 homolactic fermenters, and 6 propionate fermenters in the G group. Among them, the relative abundance of 5 butanol fermenters, 7 heterolactic fermenters, two homolactic fermenters, three propionate fermenters, as well as the total abundance of all fermenters, was significantly increased, whereas the relative abundance of one butanol fermenter and one heterolactic fermenter was significantly decreased when the diet shifted from B to G (Table 4). On the other hand, other than the unclassified genera, 10 butanol fermenters, 8 heterolactic fermenters, one homolactic fermenter, and 6 propionate fermenters were detected in the P group. Among them, the relative abundance of four butanol fermenters, four heterolactic fermenters, one homolactic fermenter, and one propionate fermenters were significantly increased, whereas the relative abundance of two butanol fermenters and two propionate fermenters was significantly decreased when the diet shifted from B to P. The total abundance of all fermenters had no significant change when the diet shifted from B to P.

### Comparison of Abundance of ETC Components and Their Major Contributors Between the Groups

As shown in Table 2, the mean abundances of subunits of Rnf, Nuo, NrfA, and Sdh were significantly increased, whereas the mean abundance of subunits of Nar was significantly decreased when the diet shifted from B to G. On the other hand, only the mean abundance of NapA subunits showed a significant increase, and the others exhibited no significantly changes when the diet shifted from B to P. Being the final step of ETC, F-type ATPase is known to be the most widely used ATP synthase in both prokaryotes and eukaryotes. In the present study, all subunits of F0-F1 ATPase were detected in the groups. The mean abundance of its subunits was 354, 769, and 383 in the B, G, and P group, respectively. Furthermore, it was significantly increased when the diet shifted from B to G, whereas no significant changes were seen when the diet shifted from B to P.

**FIGURE 3:**
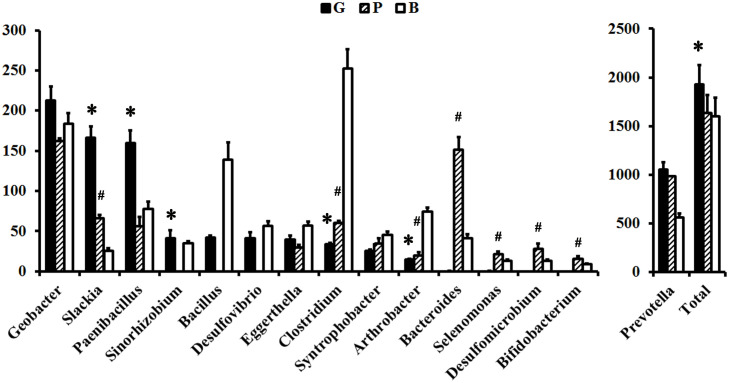
Comparisons of the relative abundance of major contributors to F-types ATPase between the groups. The relative abundance was normalized to 1,000,000 for each sample. “^∗^” indicates the significant change between the G and B groups. “#” indicates the significant change between the P and B groups.

Within the ruminal microbiome, other than the unclassified genera, 17 major contributors to Rnf, 13 major contributors to Cyd, 20 major contributors to Sdh, 7 major contributors to Nuo, 7 major contributors to NrfA, and 11 major contributors to ATPase were detected in the G group (Supplementary Table 4 and Figure 3). Among them, the abundance of 5/5/9/6/5/3 major contributors to Rnf/Cyd/Sdh/Nuo/NrfA/ATPase was significantly increased, whereas the abundance of 8/5/6/2 major contributors to Rnf/Cyd/Sdh/ATPase was significantly decreased when the diet shifted from B to G. On the other hand, other than the unclassified genera, 16 major contributors to Rnf, 10 major contributors to Cyd, 22 major contributors to Sdh, 5 major contributors to Nuo, 2 major contributors to NrfA, and 12 major contributors to ATPase were detected in the P group (Supplementary Table 4 and Figure 3). Among them, the abundance of 1/4/12/1/5 major contributor(s) of Rnf/Cyd/Sdh/Nuo/ATPase was significantly increased, whereas the abundance of 3/2/2/2 major contributor(s) of Rnf/Cyd/Sdh/ATPase was significantly decreased when the diet shifted from B to P.

## Discussion

### Effects of Dietary NFC and Protein on Rumen MCP Synthesis

In the present study, the significant increase of the rumen ammonia N was associated with the significant increase of MCP yields when the diet shifted from B to G. This is in accordance with the study of Kljak et al. (2017) and indicates that the G diet provides sufficient N and energy to the ruminal microbiome and therefore, has advantages for MCP synthesis compared with the B diet. On the other hand, rumen ammonia N was significantly increased, whereas ruminal MCP yields exhibited no significant change when the diet shifted from B to P. This result suggests that the P diet does not meet the energy requirement of ruminal microorganisms and therefore has no advantages for rumen MCP synthesis compared with the B diet. Secondly, total VFAs was significantly increased in both of G and P group compared with the B group, indicating the increase of rumen fermentation in these groups. However, the fermentation substrates that induced the increase of rumen VFAs were different in these groups. G diet majorly induced the increase of glucose fermentation, whereas P diet majorly induced the increase of protein fermentation.

### Effects of Dietary NFC and Protein on the Levels of SLP-Coupled Energy Productivity Within the Ruminal Microbiome

Our metagenomics analysis showed that (1) the total abundance of SLP enzymes was significantly increased when the diet shifted from B to G, whereas it was no significant change when the diet shifted from B to P; (2) 21 of 68 archaeal genera and 258 of 549 bacterial genera were related to the SLP within the ruminal microbiome; and (3) the relative abundance of SLP-related species was no significant change when the diet shifted from B to G, whereas it showed the significant decrease when the diet shifted from B to P. By considering the low completeness of the detected genomes, these results suggest that the NFC-rich diet provides the significant benefits with regard to the amount of SLP-related microorganisms/gene pool, whereas the protein-rich diet has no significant impact on the amount of SLP-related microorganisms/gene pool.

In the rumen, the conversion of glucose to two pyruvate molecules during glycolysis gives a net gain of two ATPs and two NADHs. Subsequently, the NADHs are oxidized to NAD^+^ by using pyruvate or one of its derivatives as an electron acceptor. This process, referred to as fermentation, may lead to the production of more ATPs, since the SLP is coupled with the oxidation of NADH in many kinds of fermentation. In the present study, four kinds of fermentation were detected within the rumen (Table 4). Among them, butanol fermentation, heterolactate fermentation, and propionate fermentation give rise to the additional ATPs (Figure 1). According to our analysis, the total abundances of NADH oxidases located on above three fermentation pathways were significantly increased when the diet shifted from B to G (1437–3054) but were no significantly changes when the diet shifted from B to P (1437–1388). This result suggests that the level of SLP-coupled energy productivity is promoted by the NFC-rich diet but not affected by the protein-rich diet in a comparison with the basal diet. Besides, a recent study has shown that an increase of intracellular NAD^+^ will promote the transfer of electrons along the ETC and thus promote the generation of ATPs in metal respiring microorganisms (Li et al., 2018). In present study, the total abundances of NADH oxidases in all fermentation pathways showed the same trends during the shift of diets, suggesting the amount of NAD^+^ and its cycling rate is promoted by the dietary NFC but not affected by the dietary protein. Accordingly, we speculate that the energy productivity of ruminal microbiome might be promoted by dietary NFC via its effects on the levels of SLP coupled to the glucose fermentation and the amount of intracellular NAD^+^.

### Effects of Dietary NFC and Protein on the Levels of ETP-Related Genes and Species Within the Ruminal Microbiome

According to our analysis, first, Ech, and cytochrome b0 were not present among the ruminal microorganisms. Second, the abundance of cytochrome c reductase (Pet and torC) and sulfate reductase Apr was less than 1 in all samples, and the species whose genomes encoded it were not detected in this study. We therefore speculated that the species whose genomes encoded cytochrome c reductase and Apr could not be present in the ruminal microbiome. Third, although the methyltransferase Met had low abundance (around 2 in all samples), it was only encoded on the genomes of archaeal methanogens that accounted for 1.2–1.4% of the ruminal microbiome. The abundances of NADH dehydrogenases Ndh and Hox were more than 10 in at least one group. However, the species whose genome encoded them were not detected. Since the completeness of bins was inadequate here, we therefore speculated that the species whose genomes encoded Met, Ndh and Hox existed in the rumen, but that their relative abundances were low. Fourth, the abundance of fumarate reductase Sdh, FADH_2_-NAD oxidoreductase Rnf, NADH dehydrogenase Nuo, nitrite reductase NrfA, and cytochrome bd complex cydA and cydB was high indicating their high abundance in the ruminal microbiome.

The microbial ETCs are diverse, and typically end up with a F-type ATPase. In present study, we evaluated the roles of ETP on the energy productivity of the ruminal microbiome by using the results from ATPase in present study, because the low completeness of received species made the detection of the integrity ETC in the specific species impossible. According to our analysis, the total mean abundance of ATPase subunits was significantly increased when the diet shifted from B to G, whereas no significant changes were seen when the diet shifted from B to P. This result indicated the ETP-coupled energy productivity was promoted by the NFC-rich diet but not affected by the protein-rich diet in a comparison with the basal diet.

The comparison on the gene abundance of the SLP enzymes and the ATPase within the groups may imply that the ETP-based ATP productivity accounts for 13–18% of SLP-based ATP productivity in the rumen microbiome. At the species level, 15 kinds of bacteria were considered to have an ETP-type energy producing mechanism within the ruminal microbiome. The detected ETC components of 15 ETP-related species are listed in Table 5. According to previous studies, *Arthrobacter*, *Geobacter*, and *Paenibacillus* are metal-respiring bacteria (Bond and Lovley, 2003;Van Driessche et al., 2005). Our analysis showed that Cyd, NrfA, Nuo, Rnf, and Sdh were used as electron transporters in these bacteria. *Clostridium* and *Eggerthella* are CO_2_-respiring acetogenic bacteria (Harris et al., 2018). Our analysis showed that Nuo, Rnf, and Sdh were commonly used as the electron transporters in them. These results are supported by previous studies showing that cytochromes, NADH dehydrogenase, NrfA, and fumarate reductase play roles in the electron transport pathway of metal respiration. Rnf and NADH dehydrogenase have important functions in the electron transport pathway of reductive acetogenesis via the Wood–Ljungdahl pathway. Next, we showed Sdh to be the common electron transporter in the sulfate-respiring *Desulfovibrio* and *Desulfomicrobium* (Kushkevych, 2014) and in the fumarate-respiring *Syntrophobacter* (Plugge et al., 2012). Rnf was shown to be the common electron transporter in the nitrate-reducing bacteria *Bacillus* and *Selenomonas* (Zhao et al., 2015; Bruna et al., 2018), the nitrogen-fixing bacterium *Sinorhizobium* (Delamuta et al., 2015), the proteolytic bacterium *Bacteroides* (Macfarlane et al., 1995), lactate-fermented bacterium *Bifidobacterium* (Macfarlane et al., 1995), and the xenobiotic-degraded bacterium *Slackia* (Cho et al., 2016). This is similar to recently discovered ETCs, all of which use Rnf as their electron transporter. In the present study, most kinds of electron transporters were detected in *Prevotella*. The high abundance and the high species diversity of this genus in the rumen might be the reason for this result. Together, these results suggest that (1) various kinds of ETCs exist in the ruminal microorganism population, and (2) ETP-type energy-producing mechanism might also play important roles in the energy productivity of the ruminal microbiome.

**Table 5:** Detected types of electron transporters in ETP-related genera.

Major contributor	Rnf	Nuo	Ndh	NrfA	Mtr	Sdh	Cyd	ATPase
*Bacillus*	Rnf							ATPase
*Selenomonas*	Rnf					Sdh		ATPase
*Sinorhizobium*	Rnf					Sdh		ATPase
*Bacteroides*	Rnf	Nuo				Sdh	Cyd	ATPase
*Arthrobacter*	Rnf						Cyd	ATPase
*Geobacter*	Rnf	Nuo				Sdh	Cyd	ATPase
*Paenibacillus*	Rnf	Nuo		NrfA		Sdh	Cyd	ATPase
*Desulfomicrobium*						Sdh	Cyd	ATPase
*Desulfovibrio*	Rnf			NrfA		Sdh		ATPase
*Clostridium*	Rnf	Nuo	Ndh			Sdh		ATPase
*Eggerthella*	Rnf	Nuo				Sdh	Cyd	ATPase
*Syntrophobacter*				NrfA		Sdh	Cyd	ATPase
*Bifidobacterium*	Rnf					Sdh	Cyd	ATPase
*Slackia*	Rnf	Nuo				Sdh	Cyd	ATPase
*Prevotella*	Rnf	Nuo		NrfA		Sdh	Cyd	ATPase


*Cyd, cytochrome bd oxidase; Mtr, methyltransferase; Ndh and Nuo, NADH dehydrogenases; NrfA, nitrite reductase; Rnf, FADH_*2*_-NAD oxidoreductase; Sdh, fumarate reductase/succinate oxidase; ATPase, F-type ATPase.*

## Conclusion

In conclusion, our data showed that, on the gene level, the mean abundance of the subunits of ATPase accounted for less than 20% of the total abundances of SLP enzymes within the ruminal microbiome. On the genus level, 279 genera were showed to be able to generate ATPs via SLP mechanism, whereas only 15 genera were showed to be able to generate ATPs via ETP mechanism within 606 detected genera. Next, an increase in dietary energy level increased rumen MCP yields, whereas an increase in dietary protein level had no significant impact on it. On the gene level, an increase in dietary energy level increased the total abundance of SLP enzymes that were coupled to the NADH oxidization in the butanol fermentation, heterolactate fermentation and propionate fermentation, and the mean abundance of F-type ATPase that catalyzed the generation of ATPs in the ETP within the ruminal microbiome, whereas an increase in dietary protein level had no significant impact on the abundances of these enzymes. On the genus level, an increase in dietary energy level increased the total abundance of 15 ETP-related genera, and the total abundance of 40 genera that own SLP-coupled fermentation pathway, whereas an increase in dietary protein level has no significant impact on the relative abundance of these genera. In summary, the comparison on the abundance of SLP enzymes and ATPase suggested that SLP occupied more important position than ETP in the energy generation within ruminal microbiome, accounting for more than 80% of total energy productivity. Furthermore, the increase in dietary energy levels promotes rumen energy productivity and MCP yield by improving levels of ETP and SLP coupled to glucose fermentation in the ruminal microbiome. But, an increase in dietary protein level has no such effects.

## Ethics Statement

This study was approved by the Animal Care and Use Committee of Nanjing Agricultural University, in compliance with the Regulations for the Administration of Affairs Concerning Experimental Animals (The State Science and Technology Commission of China, 2010).

## Author Contributions

HS wrote the manuscript. HS and ZX analyzed data. ZL and ZS designed the research. ZL and YT performed the experiments. All authors approved the final manuscript.

## Conflict of Interest Statement

The authors declare that the research was conducted in the absence of any commercial or financial relationships that could be construed as a potential conflict of interest.

## Footnotes

1
ftp://ftp.ncbi.nlm.nih.gov/genomes/refseq/
