# Corrigendum: Dietary Energy Level Promotes Rumen Microbial Protein Synthesis by Improving the Energy Productivity of the Ruminal Microbiome

**DOI:** 10.3389/fmicb.2021.770056

**Published:** 2021-09-24

**Authors:** Zhongyan Lu, Zhihui Xu, Zanming Shen, Yuanchun Tian, Hong Shen

**Affiliations:** ^1^The Key Laboratory of Animal Physiology and Biochemistry, College of Veterinary Medicine, Nanjing Agricultural University, Nanjing, China; ^2^College of Life Science, Nanjing Agricultural University, Nanjing, China; ^3^Bioinformatics Center, Nanjing Agricultural University, Nanjing, China; ^4^College of Agriculture, Nanjing Agricultural University, Nanjing, China

**Keywords:** rumen microbiome, energy productivity, substrate-level phosphorylation, electron transport phosphorylation, microbial protein synthesis, dietary modulation

There was an error in the Funding statement. The correct number for the National Natural Science Foundation of China is 31802155.

The authors apologize for this error and state that this does not change the scientific conclusions of the article in any way. The original article has been updated.

## Publisher's Note

All claims expressed in this article are solely those of the authors and do not necessarily represent those of their affiliated organizations, or those of the publisher, the editors and the reviewers. Any product that may be evaluated in this article, or claim that may be made by its manufacturer, is not guaranteed or endorsed by the publisher.

