# Supplementary material for: Dietary Energy Level Promotes Rumen Microbial Protein Synthesis by Improving the Energy Productivity of the Ruminal Microbiome
Source: Front Microbiol. 2019 Apr 17;10:847. doi: 10.3389/fmicb.2019.00847 (PMC6479175; doi:10.3389/fmicb.2019.00847)
Supplement: Supplementary file 1 [file Data_Sheet_1.PDF]

Supplementary Material

Dietary energy level promotes rumen microbial protein synthesis by improving the energy productivity of the ruminal microbiome

Zhongyan Lu[1#], Zhihui Xu[2,3#], Zanming Shen[1], Yuanchun Tian[4] and Hong Shen[2,3*]

*** Correspondence:** Hong Shen: hongshen@njau.edu.cn

1 Supplementary Figures and Tables

1.1 Supplementary Figure

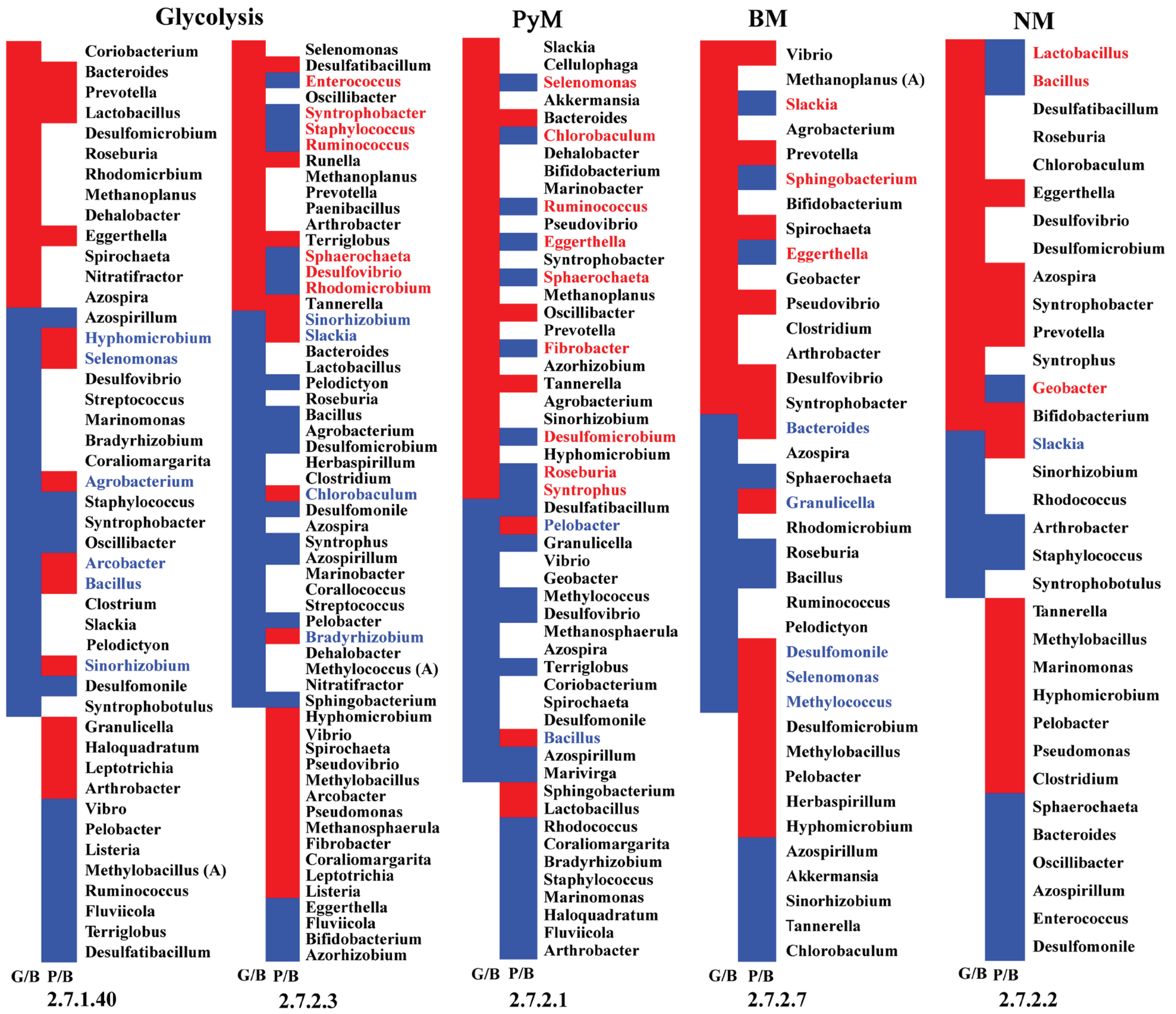

Supplementary Figure 1. Significantly changed major contributors to the specific SLP enzyme

during the shift of diets. The red box refers to the major contributors whose relative abundance was significantly increased during the shift of diets. The blue box refers to the major contributors whose relative abundance was significantly decreased during the shift of diets. The red letter refer to the major contributors whose relative abundance was significantly increased when the diet shifted from B to G and that were significant decreased when the diet shifted from B to P. The blue letters refer to the major contributors whose relative abundance was significantly decreased when the diet shifted from B to G and significantly increased when the diet shifted from B to P. G/B indicates the diet shifted from B to G. P/B indicates the diet shifted from B to P. (EC) 2.7.1.40 refers to pyruvate kinase; (EC) 2.7.2.3 refers to phosphoglycerate kinase; (EC) 2.7.2.1 refers to acetate kinase; (EC) 2.7.2.7 refers to butyrate kinase; (EC) 2.7.2.2 refers to carbamate kinase. PyM indicates the pyruvate metabolism, BM indicates butanoate metabolism and NM indicates nitrogen metabolism.

1.2 Supplementary Tables

Supplementary Table 1. EC numbers, KO number, gene annotation and coverage of enzymes located on the SLP-related and ETP-related pathways

PATHWAY	EC	KO	ENZYME	G[1]	P[1]	B[1]	G/B[2]	P/B[2]
Butanoate metabolism	1.1.1.-	K00100	bdhAB,butanol dehydrogenase	833.56±48.40	414.17±20.41	451.46±20.64	0.88	-0.12
Butanoate metabolism	1.1.1.157	K00074	paaH, hbd, fadB, mmgB; 3-hydroxybutyryl-CoA dehydrogenase	115.74±17.97	137.06±25.66	109.12±19.25	0.08	0.33
Butanoate metabolism	1.2.1.10	K04072	adhE; acetaldehyde dehydrogenase / alcohol dehydrogenase	663.37±20.90	208.92±20.27	227.83±10.88	1.54	-0.13
Butanoate metabolism	1.3.1.44	K00209	fabV, ter; enoyl-[acyl-carrier protein reductase / trans-2-enoyl-CoA reductase (NAD+)	0.62±0.01	0.62±0.01	0.97±0.00	-0.64	-0.64
Butanoate metabolism	1.3.8.1	K00248	ACADS, bcd; butyryl-CoA dehydrogenase	186.52±11.45	206.70±78.94	157.25±56.04	0.25	0.39
Butanoate metabolism	2.3.1.19	K00634	ptb; phosphate butyryltransferase	362.59±32.53	298.38±10.22	223.05±49.30	0.70	0.42
Butanoate metabolism	2.3.1.9	K00626	E2.3.1.9, atoB; acetyl-CoA C-acetyltransferase	42.67±7.90	129.26±18.39	138.22±17.43	-1.70	-0.10
Butanoate metabolism	2.3.3.10	K01641	E2.3.3.10; hydroxymethylglutaryl-CoA synthase	1.69±0.05	13.13±4.05	8.04±2.08	-2.25	0.71
Butanoate metabolism	2.7.2.7	K00929	buk; butyrate kinase	980.68±41.05	410.10±64.37	264.02±130.54	1.89	0.64
Butanoate metabolism	2.8.3.5	K01027	OXCT; 3-oxoacid CoA-transferase	1.72±0.01	2.66±0.01	2.54±0.00	-0.56	0.07
Butanoate metabolism	2.8.3.5	K01028	E2.8.3.5A, scoA; 3-oxoacid CoA-transferase subunit A	2.71±0.02	2.63±0.02	2.74±0.00	-0.01	-0.05
Butanoate metabolism	2.8.3.5	K01029	E2.8.3.5B, scoB; 3-oxoacid CoA-transferase subunit B	55.72±4.06	53.05±4.31	37.59±2.20	0.57	0.50
Butanoate metabolism	2.8.3.8	K01034	atoD; acetate CoA/acetoacetate CoA-transferase alpha subunit	50.32±10.28	93.36±11.16	52.91±10.04	-0.07	0.82
Butanoate metabolism	2.8.3.8	K01035	atoA; acetate CoA/acetoacetate CoA-transferase beta subunit	3.76±0.04	13.19±0.03	9.35±0.00	-1.31	0.50
Butanoate metabolism	2.8.3.8	K19709	ydiF; acetate CoA-transferase	16.85±2.01	19.45±1.60	18.11±4.02	-0.10	0.10
Butanoate metabolism	4.1.3.4	K01640	E4.1.3.4, HMGCL, hmgL; hydroxymethylglutaryl-CoA lyase	8.90±1.31	22.64±1.78	24.70±2.38	-1.47	-0.13
Butanoate metabolism	4.2.1.17	K01782	fadJ; 3-hydroxyacyl-CoA dehydrogenase / enoyl-CoA hydratase / 3-hydroxybutyryl-CoA epimerase	6.32±2.18	2.56±0.84	1.98±0.13	1.68	0.37
Butanoate metabolism	6.2.1.16	K01907	AACS, acsA; acetoacetyl-CoA synthetase	1.47±0.57	1.12±0.82	3.57±1.60	-1.28	-1.68
Butanoate metabolism	6.2.1.2	K01896	ACSM; medium-chain acyl-CoA synthetase	5.01±1.84	12.17±3.03	11.08±1.54	-1.14	0.14
Entner Doudoroff pathway	1.1.1.47	K00034	gdh; glucose 1-dehydrogenase	5.14±2.05	29.56±9.25	12.12±2.41	-1.24	1.29
Entner Doudoroff pathway	2.7.1.12	K00851	E2.7.1.12, gntK, idnK; gluconokinase	7.19±1.90	19.86±3.57	12.61±2.67	-0.81	0.66
Entner Doudoroff pathway	3.1.1.17	K01053	E3.1.1.17, gnl, RGN; gluconolactonase	65.83±2.76	15.08±3.38	33.02±2.20	1.00	-1.13
Entner Doudoroff pathway	4.1.2.14 4.1.3.42	K01625	eda; 2-dehydro-3-deoxyphosphogluconate aldolase / (4S)-4-hydroxy-2-oxoglutarate aldolase	351.87±38.58	430.74±55.25	464.09±61.49	-0.40	-0.11

Entner Doudoroff pathway	4.2.1.12	K01690	edd; phosphogluconate dehydratase	0.01±0.00	0.04±0.00	0.02±0.00	-1.17	1.14
Glycolysis	1.2.1.12	K00134	GAPDH, gapA; glyceraldehyde 3-phosphate dehydrogenase	725.73±79.53	979.72±101.46	1078.04±123.99	-0.57	-0.14
Glycolysis	1.2.1.59	K00150	gap2; glyceraldehyde-3-phosphate dehydrogenase (NAD(P))	6.81±1.23	24.67±2.67	19.29±1.91	-1.50	0.36
Glycolysis	2.7.1.40	K00873	PK, pyk; pyruvate kinase	589.59±25.46	480.02±39.72	436.98±43.85	0.43	0.14
Glycolysis	2.7.2.3	K00927	PGK, pgk; phosphoglycerate kinase	1490.58±76.36	1097.76±26.08	1158.74±67.61	0.36	-0.08
Glycolysis	2.7.9.1	K01006	ppdK; pyruvate, orthophosphate dikinase	1610.97±136.50	1393.32±140.10	1278.26±129.71	0.33	0.12
Glycolysis	2.7.9.2	K01007	pps, ppsA; pyruvate, water dikinase	16.19±4.16	29.49±9.99	14.45±2.27	0.16	1.03
Glycolysis	4.1.1.32	K01596	E4.1.1.32, pckA, PCK; phosphoenolpyruvate carboxykinase (GTP)	39.31±5.67	21.96±4.10	42.69±6.46	-0.12	-0.96
Glycolysis	4.1.1.49	K01610	E4.1.1.49, pckA; phosphoenolpyruvate carboxykinase (ATP)	1057.42±110.06	797.06±86.24	774.40±68.16	0.45	0.04
Glycolysis	4.2.1.11	K01689	ENO, eno; enolase	557.59±45.91	766.48±69.97	699.25±45.36	-0.33	0.13
Glycolysis	5.4.2.11	K01834	PGAM, gpmA; 2,3-bisphosphoglycerate-dependent phosphoglycerate mutase	172.30±44.37	247.44±17.52	225.17±21.74	-0.39	0.14
Glycolysis	5.4.2.12	K15633	gpmI; 2,3-bisphosphoglycerate-independent phosphoglycerate mutase	1441.19±88.64	721.93±26.06	762.39±61.56	0.92	-0.08
Glycolysis	5.4.2.12	K15634	gpmB; probable phosphoglycerate mutase	36.54±7.24	42.99±4.44	55.69±5.37	-0.61	-0.37
Glycolysis	5.4.2.12	K15635	apgM; 2,3-bisphosphoglycerate-independent phosphoglycerate mutase	213.31±36.53	131.01±29.49	163.24±17.83	0.39	-0.32
Glycolysis	6.2.1.1	K01895	ACSS, acs; acetyl-CoA synthetase	544.65±44.10	377.79±13.91	319.32±85.79	0.77	0.24
Glycolysis	6.2.1.13	K01905	E6.2.1.13; acetyl-CoA synthetase (ADP-forming)	3.52±1.29	9.96±2.72	8.42±3.26	-1.26	0.24
Methane metabolism	1.12.98.1	K00441	frhB; coenzyme F420 hydrogenase subunit beta	2.27±0.47	5.30±3.85	4.21±0.99	-0.89	0.33
Methane metabolism	1.12.98.2	K13942	hmd; 5,10-methenyltetrahydromethanopterin hydrogenase	2.33±0.47	0.79±0.08	1.18±0.38	0.98	-0.57
Methane metabolism	1.2.7.12	K00205	fwdF, fmdF; 4Fe-4S ferredoxin	4.56±1.20	27.79±4.12	11.39±3.64	-1.32	1.29
Methane metabolism	1.2.7.12	K11260	fwdG; 4Fe-4S ferredoxin	1.60±0.89	34.31±2.10	3.07±0.82	-0.94	3.48
Methane metabolism	1.5.98.1	K00319	mtd; methylenetetrahydromethanopterin dehydrogenase	3.02±0.40	113.61±22.46	5.42±2.27	-0.84	4.39
Methane metabolism	1.5.98.2	K00320	mer; 5,10-methylenetetrahydromethanopterin reductase	1.60±0.23	21.53±3.72	2.58±0.64	-0.69	3.06
Methane metabolism	1.8.98.1	K03388	hdrA; heterodisulfide reductase subunit A	31.38±9.27	42.63±7.46	28.96±1.91	0.12	0.56
Methane metabolism	1.8.98.1	K03389	hdrB; heterodisulfide reductase subunit B	2.62±0.99	11.86±6.28	8.47±1.51	-1.69	0.49
Methane metabolism	1.8.98.1	K03390	hdrC; heterodisulfide reductase subunit C	28.21±8.49	52.37±4.32	37.06±4.93	-0.39	0.50
Methane metabolism	2.3.1.101	K00672	ftr; formylmethanofuran--tetrahydromethanopterin N-formyltransferase	3.65±0.58	37.66±8.61	4.45±0.93	-0.28	3.08
Methane metabolism	2.8.4.1	K00401	mcrB; methyl-coenzyme M reductase beta subunit	3.07±0.62	6.79±0.53	5.50±0.84	-0.84	0.30
Methane metabolism	3.5.4.27	K01499	mch; methenyltetrahydromethanopterin cyclohydrolase	0.70±0.15	5.99±2.69	0.92±0.22	-0.40	2.70
Nitrogen metabolism	1.18.6.1	K02586	nifD; nitrogenase molybdenum-iron protein alpha chain	0.13±0.01	0.02±0.01	0.08±0.00	0.71	-1.96
Nitrogen metabolism	1.18.6.1	K02588	nifH; nitrogenase iron protein NifH	34.83±5.57	24.15±3.18	32.03±3.84	0.12	-0.41
Nitrogen metabolism	1.18.6.1	K02591	nifK; nitrogenase molybdenum-iron protein beta chain	0.00±0.00	0.00±0.00	0.01±0.00	G0	-1.42

Nitrogen metabolism	1.4.1.13 1.4.1.14	K00264	GLT1; glutamate synthase (NADPH/NADH)	17.86±4.47	25.18±5.43	14.91±5.17	0.26	0.76
Nitrogen metabolism	1.4.1.13 1.4.1.14	K00265	gltB; glutamate synthase (NADPH/NADH) large chain	1230.81±133.30	1053.23±169.46	823.00±81.62	0.58	0.36
Nitrogen metabolism	1.4.1.13 1.4.1.14	K00266	gltD; glutamate synthase (NADPH/NADH) small chain	1786.05±140.49	1443.70±215.75	1375.74±190.19	0.38	0.07
Nitrogen metabolism	1.4.1.2	K00260	gudB, rocG; glutamate dehydrogenase	1.92±0.39	6.26±2.16	10.31±2.11	-2.42	-0.72
Nitrogen metabolism	1.4.1.3	K00261	GLUD1 2, gdhA; glutamate dehydrogenase (NAD(P)+)	2.79±0.38	24.99±1.67	14.43±3.02	-2.37	0.79
Nitrogen metabolism	1.4.1.4	K00262	gdhA; glutamate dehydrogenase (NADP+)	1291.51±123.88	1008.36±142.13	1150.75±256.47	0.17	-0.19
Nitrogen metabolism	1.4.7.1	K00284	glutamate synthase (ferredoxin)	19.83±5.82	39.74±3.64	19.69±2.70	0.01	1.01
Nitrogen metabolism	1.7.7.1	K00366	nirA; ferredoxin-nitrite reductase	0.25±0.04	2.99±0.02	1.88±0.00	-2.91	0.67
Nitrogen metabolism	1.7.7.2	K00367	narB; ferredoxin-nitrate reductase	6.38±1.71	37.21±9.18	4.13±1.06	0.63	3.17
Nitrogen metabolism	2.7.2.2	K00926	arcC; carbamate kinase	202.21±10.26	174.72±46.94	152.81±28.83	0.40	0.19
Nitrogen metabolism	4.2.1.1	K01673	cynT, can; carbonic anhydrase	541.39±50.02	421.56±27.93	383.34±50.39	0.50	0.14
Nitrogen metabolism	6.3.1.2	K01915	glnA, GLUL; glutamine synthetase	1978.42±168.58	1563.47±172.76	1460.30±114.63	0.44	0.10
Nitrogen metabolism	6.3.4.16	K01948	CPS1; carbamoyl-phosphate synthase (ammonia)	0.43±0.14	1.57±0.92	3.45±0.82	-3.01	-1.14
Pentose phosphate pathway	1.1.1.343	K00033	PGD, gnd, gntZ; 6-phosphogluconate dehydrogenase	49.07±7.30	145.82±16.96	124.39±22.19	-1.34	0.23
Pentose phosphate pathway	1.1.1.363	K00036	G6PD, zwf; glucose-6-phosphate 1-dehydrogenase	0.00±0.00	0.30±0.05	0.97±0.08	G0	-1.68
Pentose phosphate pathway	1.1.1.44	K00033	PGD, gnd, gntZ; 6-phosphogluconate dehydrogenase	49.07±7.30	145.82±16.96	124.39±22.19	-1.34	0.23
Pentose phosphate pathway	1.1.1.49	K00036	G6PD, zwf; glucose-6-phosphate 1-dehydrogenase	0.00±0.00	0.30±0.05	0.97±0.08	G0	-1.68
Pentose phosphate pathway	3.1.1.31	K07404	pgl; 6-phosphogluconolactonase	17.67±1.27	19.47±2.60	29.88±3.07	-0.76	-0.62
Propanoate metabolism	1.2.7.1	K00169	porA; pyruvate ferredoxin oxidoreductase alpha subunit	15.89±2.73	32.28±7.03	37.42±7.29	-1.24	-0.21
Propanoate metabolism	1.2.7.1	K00170	porB; pyruvate ferredoxin oxidoreductase beta subunit	15.47±2.75	29.88±2.91	36.65±7.46	-1.24	-0.29
Propanoate metabolism	1.2.7.1	K00171	porD; pyruvate ferredoxin oxidoreductase delta subunit	18.04±2.85	25.93±1.65	23.11±6.86	-0.36	0.17
Propanoate metabolism	1.2.7.1	K00172	porG; pyruvate ferredoxin oxidoreductase gamma subunit	16.79±2.46	26.96±4.81	41.33±6.07	-1.30	-0.62
Propanoate metabolism	2.3.1.54	K00656	E2.3.1.54, pflD; formate C-acetyltransferase	209.99±26.87	260.39±33.44	263.91±42.23	-0.33	-0.02
Propanoate metabolism	2.3.1.8	K00625	E2.3.1.8, pta; phosphate acetyltransferase	518.53±42.84	309.88±31.48	310.94±33.25	0.74	0.00
Propanoate metabolism	2.3.1.8	K13788	pta; phosphate acetyltransferase	35.58±2.13	27.02±8.12	43.44±4.13	-0.29	-0.69
Propanoate metabolism	2.7.2.1	K00925	ackA; acetate kinase	910.36±120.20	625.56±57.83	749.60±63.99	0.28	-0.26
Propanoate metabolism	2.8.3.1	K01026	pct; propionate CoA-transferase	1.07±0.20	4.58±1.32	2.17±1.37	-1.03	1.08
Propanoate metabolism	6.2.1.1	K01895	ACSS, acs; acetyl-CoA synthetase	544.65±44.10	377.79±55.63	319.32±154.43	0.77	0.24
Propanoate metabolism	6.2.1.3	K01897	ACSL, fadD; long-chain acyl-CoA synthetase	2021.10±161.38	2142.94±282.78	2128.68±204.43	-0.07	0.01
Propanoate metabolism	6.2.1.3	K15013	ACSBG; long-chain-fatty-acid--CoA ligase ACSBG	1.69±0.69	41.39±3.63	34.32±5.15	-4.34	0.27
Pyruvate metabolism	1.1.1.1	K00001	E1.1.1.1, adh; alcohol dehydrogenase	8.15±1.77	5.66±1.65	3.87±1.31	1.07	0.55
Pyruvate metabolism	1.1.1.1	K00121	frmA, ADH5, adhC; S-(hydroxymethyl)glutathione dehydrogenase / alcohol	16.55±6.07	2.01±1.40	6.49±1.48	1.35	-1.69

			dehydrogenase					
Pyruvate metabolism	1.1.1.1	K04072	adhE; acetaldehyde dehydrogenase / alcohol dehydrogenase	269.41±20.90	137.84±20.27	227.83±54.40	0.24	-0.73
Pyruvate metabolism	1.1.1.1	K13953	adhP; alcohol dehydrogenase, propanol-preferring	14.74±2.87	4.75±1.14	4.29±1.33	1.78	0.15
Pyruvate metabolism	1.1.1.1	K13954	yiaY; alcohol dehydrogenase	121.40±10.44	32.83±5.67	38.95±7.32	1.64	-0.25
Pyruvate metabolism	1.1.1.2	K00002	AKR1A1, adh; alcohol dehydrogenase (NADP+)	79.88±11.90	81.79±17.51	78.46±18.96	0.03	0.06
Pyruvate metabolism	1.1.1.27	K00016	LDH, ldh; L-lactate dehydrogenase	118.76±33.54	125.58±27.77	86.46±15.35	0.46	0.54
Pyruvate metabolism	1.1.1.37	K00024	mdh; malate dehydrogenase	612.69±54.98	415.07±96.21	383.46±55.67	0.68	0.11
Pyruvate metabolism	1.2.1.-	K00138	aldB; aldehyde dehydrogenase	0.85±0.02	3.76±0.03	2.78±0.00	-1.71	0.43
Pyruvate metabolism	1.2.1.3	K00128	ALDH; aldehyde dehydrogenase (NAD+)	178.16±12.41	218.19±21.06	231.15±40.24	-0.38	-0.08
Pyruvate metabolism	1.2.1.3	K00149	ALDH9A1; aldehyde dehydrogenase family 9 member A1	25.84±6.17	1.57±0.47	28.42±9.31	-0.14	-4.18
Pyruvate metabolism	1.2.1.5	K00129	E1.2.1.5; aldehyde dehydrogenase (NAD(P)+)	2.67±0.71	0.13±0.06	0.19±0.09	3.82	-0.52
Pyruvate metabolism	1.2.4.1	K00161	PDHA, pdhA; pyruvate dehydrogenase E1 component alpha subunit	19.98±3.73	16.08±4.88	17.09±4.44	0.23	-0.09
Pyruvate metabolism	1.2.4.1	K00162	PDHB, pdhB; pyruvate dehydrogenase E1 component beta subunit	35.40±8.08	24.58±4.82	45.11±10.76	-0.35	-0.88
Pyruvate metabolism	1.2.7.1	K00169	porA; pyruvate ferredoxin oxidoreductase alpha subunit	15.89±2.73	32.28±10.55	37.42±7.29	-1.24	-0.21
Pyruvate metabolism	1.2.7.1	K00170	porB; pyruvate ferredoxin oxidoreductase beta subunit	15.47±2.75	29.88±4.36	36.65±7.46	-1.24	-0.29
Pyruvate metabolism	1.2.7.1	K00171	porD; pyruvate ferredoxin oxidoreductase delta subunit	18.04±2.85	25.93±13.20	23.11±6.86	-0.36	0.17
Pyruvate metabolism	1.2.7.1	K00172	porG; pyruvate ferredoxin oxidoreductase gamma subunit	16.79±2.46	26.96±4.81	41.33±10.12	-1.30	-0.62
Pyruvate metabolism	1.2.7.1	K03737	por, nifJ; pyruvate-ferredoxin/flavodoxin oxidoreductase	2753.87±313.32	2417.58±245.67	2512.23±334.14	0.13	-0.06
Pyruvate metabolism	1.2.7.11	K00174	korA, oorA, oforA; 2-oxoglutarate/2-oxoacid ferredoxin oxidoreductase subunit alpha	1649.51±115.85	699.85±59.78	651.54±64.79	1.34	0.10
Pyruvate metabolism	1.2.7.11	K00175	korB, oorB, oforB; 2-oxoglutarate/2-oxoacid ferredoxin oxidoreductase subunit beta	1268.16±180.16	613.71±98.42	616.62±174.35	1.04	-0.01
Pyruvate metabolism	1.8.1.4	K00382	DLD, lpd, pdhD; dihydrolipoamide dehydrogenase	629.51±106.28	432.19±53.83	514.41±107.07	0.29	-0.25
Pyruvate metabolism	2.3.1.12	K00627	DLAT, aceF, pdhC; pyruvate dehydrogenase E2 component (dihydrolipoamide acetyltransferase)	44.83±4.81	19.77±5.45	34.90±5.48	0.36	-0.82
Pyruvate metabolism	2.3.1.54	K00656	E2.3.1.54, pflD; formate C-acetyltransferase	209.99±26.87	260.39±26.75	263.91±33.78	-0.33	-0.02
Pyruvate metabolism	2.3.1.8	K00625	E2.3.1.8, pta; phosphate acetyltransferase	518.53±42.84	309.88±31.48	310.94±33.25	0.74	0.00
Pyruvate metabolism	2.3.1.8	K13788	pta; phosphate acetyltransferase	35.58±2.13	27.02±5.41	43.44±8.27	-0.29	-0.69
Pyruvate metabolism	2.7.2.1	K00925	ackA; acetate kinase	910.36±60.10	625.56±28.92	749.60±42.66	0.28	-0.26
Pyruvate metabolism	4.2.1.2	K01676	E4.2.1.2A, fumA, fumB; fumarate hydratase, class I	876.80±79.00	534.93±56.37	451.69±64.04	0.96	0.24
Pyruvate metabolism	4.2.1.2	K01677	E4.2.1.2AA, fumA; fumarate hydratase subunit alpha	61.38±4.84	51.96±4.17	77.84±11.77	-0.34	-0.58
Pyruvate metabolism	4.2.1.2	K01678	E4.2.1.2AB, fumB; fumarate hydratase subunit beta	57.15±6.64	49.94±9.16	79.16±8.82	-0.47	-0.66
Pyruvate metabolism	4.2.1.2	K01679	E4.2.1.2B, fumC; fumarate hydratase, class II	167.27±25.01	225.01±20.49	223.92±35.98	-0.42	0.01
Pyruvate metabolism	6.4.1.1	K01958	PC, pyc; pyruvate carboxylase	74.76±4.17	40.21±12.50	60.87±14.13	0.30	-0.60

Pyruvate metabolism	6.4.1.1	K01960	pycB; pyruvate carboxylase subunit B	852.31±72.87	647.93±50.83	691.57±74.29	0.30	-0.09
Pyruvate metabolism	EutG	K04022	eutG; alcohol dehydrogenase	40.05±5.12	13.59±3.35	12.94±3.21	1.63	0.07
Reductive acetyl-CoA pathway	1.2.7.4	K00192	cdhA; acetyl-CoA decarbonylase/synthase complex subunit alpha	0.56±0.07	3.86±0.17	1.54±0.76	-1.47	1.32
Reductive acetyl-CoA pathway	1.5.1.20	K00297	metF, MTHFR; methylenetetrahydrofolate reductase (NADPH)	531.42±36.42	273.44±39.57	280.66±75.87	0.92	-0.04
Reductive acetyl-CoA pathway	1.5.1.5	K00288	MTHFD; methylenetetrahydrofolate dehydrogenase (NADP+) / methenyltetrahydrofolate cyclohydrolase / formyltetrahydrofolate synthetase	27.31±3.92	10.32±3.72	19.28±3.69	0.50	-0.90
Reductive acetyl-CoA pathway	1.5.1.5	K01491	folD; methylenetetrahydrofolate dehydrogenase (NADP+) / methenyltetrahydrofolate cyclohydrolase	591.50±57.43	766.44±116.07	806.74±142.81	-0.45	-0.07
Reductive acetyl-CoA pathway	2.1.1.245	K00197	cdhE, acsC; acetyl-CoA decarbonylase/synthase complex subunit gamma	1.55±0.40	23.39±5.62	4.33±1.85	-1.49	2.43
Reductive acetyl-CoA pathway	6.3.4.3	K00288	MTHFD; methylenetetrahydrofolate dehydrogenase (NADP+) / methenyltetrahydrofolate cyclohydrolase / formyltetrahydrofolate synthetase	27.31±7.85	10.32±4.96	19.28±3.69	0.50	-0.90
Reductive acetyl-CoA pathway	6.3.4.3	K01938	fhs; formate--tetrahydrofolate ligase	384.00±25.27	491.13±38.65	703.60±83.22	-0.87	-0.52
Sulfur metabolism	1.8.1.2	K00380	cysJ; sulfite reductase (NADPH) flavoprotein alpha-component	0.00±0.00	0.04±0.00	0.01±0.00	G0	1.54
Sulfur metabolism	1.8.4.8	K00390	cysH; phosphoadenosine phosphosulfate reductase	39.13±8.35	42.27±4.88	38.78±13.25	0.01	0.12
Sulfur metabolism	1.8.7.1	K00392	sir; sulfite reductase (ferredoxin)	0.00±0.00	0.80±0.27	0.48±0.12	G0	0.72
Sulfur metabolism	2.3.1.30	K00640	cysE; serine O-acetyltransferase	726.87±51.77	605.91±47.57	794.21±50.82	-0.13	-0.39
Sulfur metabolism	2.3.1.46	K00651	metA; homoserine O-succinyltransferase	370.88±28.55	215.54±31.79	236.92±60.40	0.65	-0.14
Sulfur metabolism	2.5.1.-	K10764	metZ; O-succinylhomoserine sulfhydrylase	0.50±0.08	6.11±0.36	1.06±0.20	-1.09	2.53
Sulfur metabolism	2.5.1.47	K01738	cysK; cysteine synthase A	963.00±53.80	1053.25±102.06	1212.20±114.02	-0.33	-0.20
Sulfur metabolism	2.5.1.47	K12339	cysM; cysteine synthase B	0.05±0.01	1.34±0.78	2.67±0.14	-5.74	-0.99
Sulfur metabolism	2.5.1.47	K17069	MET17; O-acetylhomoserine/O-acetylserine sulfhydrylase	16.03±3.40	4.12±1.39	2.22±0.80	2.85	0.89
Sulfur metabolism	2.5.1.48	K01739	metB; cystathionine gamma-synthase	16.98±4.33	14.06±2.74	16.80±2.39	0.02	-0.26
Sulfur metabolism	2.7.1.25	K00860	cysC; adenylylsulfate kinase	13.86±4.70	5.66±1.57	13.55±4.75	0.03	-1.26
Sulfur metabolism	2.7.7.4	K00955	cysNC; bifunctional enzyme CysN/CysC	0.32±0.03	0.08±0.01	0.04±0.00	3.07	1.03
Sulfur metabolism	2.7.7.4	K00956	cysN; sulfate adenylyltransferase subunit 1	8.95±2.27	13.02±5.51	15.25±4.24	-0.77	-0.23
Sulfur metabolism	2.7.7.4	K00957	cysD; sulfate adenylyltransferase subunit 2	8.26±1.06	14.04±2.99	16.99±3.82	-1.04	-0.27
Sulfur metabolism	2.7.7.4	K00958	sat, met3; sulfate adenylyltransferase	2.10±1.28	2.81±2.78	4.53±2.15	-1.11	-0.69
Sulfur metabolism	4.4.1.2	K17217	mccB; cystathionine gamma-lyase / homocysteine desulfhydrase	0.91±0.04	0.60±0.07	0.15±0.05	2.63	2.02
ATPase	ATPF1A	K02111	F-type H+-transporting ATPase subunit alpha	1054.30±126.45	388.58±67.47	380.58±61.31	1.47	0.03
ATPase	ATPF1B	K02112	F-type H+-transporting ATPase subunit beta	896.35±76.10	559.47±43.28	493.84±50.74	0.86	0.18
ATPase	ATPF1G	K02115	F-type H+-transporting ATPase subunit gamma	1258.48±129.87	612.03±102.57	629.24±78.14	1.00	-0.04
ATPase	ATPF1D	K02113	F-type H+-transporting ATPase subunit delta	404.81±58.10	196.87±42.95	208.09±23.73	0.96	-0.08
ATPase	ATPF1E	K02114	F-type H+-transporting ATPase subunit epsilon	520.84±42.38	239.63±34.83	193.30±22.60	1.43	0.31
ATPase	ATPF0A	K02108	F-type H+-transporting ATPase subunit a	804.66±61.20	422.33±48.25	428.23±30.59	0.91	-0.02

ATPase	ATPF0B	K02109	F-type H+-transporting ATPase subunit b	493.42±67.79	316.63±48.04	269.97±20.90	0.87	0.23
ATPase	ATPF0C	K02110	F-type H+-transporting ATPase subunit c	717.20±64.81	325.44±30.87	225.38±47.33	1.67	0.53
FADH2-NAD oxidoreductase	RnfA	K03617	electron transport complex protein RnfA	1513.99±379.66	422.33±69.02	474.79±51.49	1.67	-0.17
FADH3-NAD oxidoreductase	RnfB	K03616	electron transport complex protein RnfB	615.30±44.98	410.33±53.28	449.13±42.60	0.45	-0.13
FADH4-NAD oxidoreductase	RnfC	K03615	electron transport complex protein RnfC	812.66±61.58	472.78±71.45	526.88±73.19	0.63	-0.16
FADH5-NAD oxidoreductase	RnfD	K03614	electron transport complex protein RnfD	771.83±49.96	412.60±67.51	403.69±45.79	0.94	0.03
FADH6-NAD oxidoreductase	RnfE	K03613	electron transport complex protein RnfE	1283.16±169.21	460.10±84.43	482.61±41.54	1.41	-0.07
FADH7-NAD oxidoreductase	RnfG	K03612	electron transport complex protein RnfG	463.29±61.28	325.37±83.78	321.63±39.08	0.53	0.02
Methanogenesis	MtrC	K00579	tetrahydromethanopterin S-methyltransferase subunit C	2.25±0.56	2.23±0.46	1.91±0.29	0.23	0.22
Methanogenesis	MtrH	K00584	tetrahydromethanopterin S-methyltransferase subunit H	2.08±0.74	1.37±0.30	1.82±0.18	0.19	-0.40
Methanogenesis	MtrD	K00580	tetrahydromethanopterin S-methyltransferase subunit D	2.65±0.25	1.18±0.46	2.06±0.30	0.37	-0.81
Methanogenesis	MtrE	K00581	tetrahydromethanopterin S-methyltransferase subunit E	0.49±0.08	0.87±0.15	0.72±0.24	-0.53	0.28
Methanogenesis	MtrA	K00577	tetrahydromethanopterin S-methyltransferase subunit A	3.55±1.19	4.17±1.70	3.97±0.75	-0.16	0.07
NADH dehydrogenase	NuoA	K00330	NADH-quinone oxidoreductase subunit A	247.15±22.47	120.94±20.18	113.00±11.59	1.13	0.10
NADH dehydrogenase	NuoB	K00331	NADH-quinone oxidoreductase subunit B	306.32±61.54	132.78±34.78	137.88±28.27	1.15	-0.05
NADH dehydrogenase	NuoC	K00332	NADH-quinone oxidoreductase subunit C	1.46±0.55	0.19±0.04	0.29±0.06	2.33	-0.60
NADH dehydrogenase	NuoD	K00333	NADH-quinone oxidoreductase subunit D	21.87±7.86	9.92±1.10	13.21±2.44	0.73	-0.41
NADH dehydrogenase	NuoE	K00334	NADH-quinone oxidoreductase subunit E	47.19±6.56	35.52±2.02	43.86±5.26	0.11	-0.30
NADH dehydrogenase	NuoF	K00335	NADH-quinone oxidoreductase subunit F	131.04±29.90	87.57±10.21	108.78±25.33	0.27	-0.31
NADH dehydrogenase	NuoG	K00336	NADH-quinone oxidoreductase subunit G	19.23±6.93	11.13±2.85	11.13±3.35	0.79	0.00
NADH dehydrogenase	NuoH	K00337	NADH-quinone oxidoreductase subunit H	278.69±41.33	139.21±14.35	140.44±17.06	0.99	-0.01
NADH dehydrogenase	NuoI	K00338	NADH-quinone oxidoreductase subunit I	218.99±85.86	91.69±37.51	85.24±19.81	1.36	0.11
NADH dehydrogenase	NuoJ	K00339	NADH-quinone oxidoreductase subunit J	220.70±65.60	137.23±23.59	132.62±37.35	0.73	0.05
NADH dehydrogenase	NuoK	K00340	NADH-quinone oxidoreductase subunit K	277.67±33.35	162.67±38.13	127.11±29.16	1.13	0.36
NADH dehydrogenase	NuoL	K00341	NADH-quinone oxidoreductase subunit L	439.91±42.71	334.29±25.18	266.75±46.27	0.72	0.33
NADH dehydrogenase	NuoM	K00342	NADH-quinone oxidoreductase subunit M	586.51±51.17	270.36±26.10	235.98±41.23	1.31	0.20
NADH dehydrogenase	NuoN	K00343	NADH-quinone oxidoreductase subunit N	429.49±79.48	147.72±13.67	172.50±11.14	1.32	-0.22
NADH dehydrogenase	NdhA	K05572	NAD(P)H-quinone oxidoreductase subunit 1	17.16±4.44	12.36±3.49	9.10±3.16	0.91	0.44
NADH dehydrogenase	NdhB	K05573	NAD(P)H-quinone oxidoreductase subunit 2	50.17±7.25	29.76±4.15	27.12±4.91	0.89	0.13
NADH dehydrogenase	NdhC	K05574	NAD(P)H-quinone oxidoreductase subunit 3	8.96±1.35	10.67±2.32	8.00±3.28	0.16	0.42

NADH dehydrogenase	NdhD	K05575	NAD(P)H-quinone oxidoreductase subunit 4	7.91±1.63	5.48±1.44	5.41±1.02	0.55	0.02
NADH dehydrogenase	NdhE	K05576	NAD(P)H-quinone oxidoreductase subunit 4L	28.34±5.64	36.30±7.63	30.98±6.04	-0.13	0.23
NADH dehydrogenase	NdhF	K05577	NAD(P)H-quinone oxidoreductase subunit 5	6.44±0.90	8.70±1.71	7.90±2.13	-0.30	0.14
NADH dehydrogenase	NdhG	K05578	NAD(P)H-quinone oxidoreductase subunit 6	4.65±1.55	5.06±1.44	5.00±1.84	-0.11	0.02
NADH dehydrogenase	NdhH	K05579	NAD(P)H-quinone oxidoreductase subunit H	2.79±0.76	4.57±1.98	2.66±0.80	0.07	0.78
NADH dehydrogenase	NdhI	K05580	NAD(P)H-quinone oxidoreductase subunit I	9.38±2.02	8.07±3.60	6.45±2.08	0.54	0.32
NADH dehydrogenase	NdhJ	K05581	NAD(P)H-quinone oxidoreductase subunit J	8.13±1.77	10.21±2.62	7.46±1.46	0.12	0.45
NADH dehydrogenase	NdhK	K05582	NAD(P)H-quinone oxidoreductase subunit K	7.64±1.14	11.52±3.30	6.32±1.05	0.27	0.87
NADH dehydrogenase	HoxE	K05586	bidirectional [NiFe] hydrogenase diaphorase subunit	14.56±2.30	3.80±1.06	8.23±1.68	0.82	-1.11
NADH dehydrogenase	HoxF	K05587	bidirectional [NiFe] hydrogenase diaphorase subunit	14.42±3.20	12.81±2.18	16.17±3.51	-0.16	-0.34
NADH dehydrogenase	HoxU	K05588	bidirectional [NiFe] hydrogenase diaphorase subunit	12.25±2.19	5.96±1.45	8.27±1.94	0.57	-0.47
Cytochrome c reductase	PetC/CYC1	K00413	ubiquinol-cytochrome c reductase cytochrome c1 subunit	0.20±0.05	0.39±0.03	0.30±0.07	-0.57	0.38
Cytochrome c reductase	PetB/CYTB	K00412	ubiquinol-cytochrome c reductase cytochrome b subunit	1.53±0.51	1.75±0.14	1.13±0.24	0.45	0.64
Cytochrome c reductase	PetA/UQCRFS	K00411	ubiquinol-cytochrome c reductase iron-sulfur subunit	0.37±0.09	0.54±0.04	0.46±0.04	-0.32	0.25
Cytochrome c reductase	torC	K03532	trimethylamine-N-oxide reductase (cytochrome c), cytochrome c-type subunit TorC	0.47±0.03	0.83±0.04	0.65±0.07	-0.45	0.36
Cytochrome bd oxidase	cydA	K00425	cytochrome bd ubiquinol oxidase subunit I	386.71±50.13	585.43±33.78	390.79±37.46	-0.02	0.58
Cytochrome bd oxidase	cydB	K00426	cytochrome bd ubiquinol oxidase subunit II	437.60±47.13	493.58±40.41	341.73±36.08	0.36	0.53
Sulfate reductase	AprA	K00394	adenylylsulfate reductase, subunit A	0.60±0.03	0.49±0.05	0.65±0.05	-0.12	-0.41
Sulfate reductase	AprB	K00395	adenylylsulfate reductase, subunit B	0.68±0.06	0.70±0.09	0.91±0.05	-0.41	-0.37
Nitrite reductase	NrfA	K03385	nitrite reductase (cytochrome c-552)	403.53±65.93	202.95±29.52	176.38±24.09	1.19	0.20
Nitrate reductase	NarH	K00371	nitrate reductase / nitrite oxidoreductase, beta subunit	0.37±0.05	1.11±0.10	1.88±0.22	-2.35	-0.76
Nitrate reductase	NarI	K00374	nitrate reductase gamma subunit	0.00±0.00	0.19±0.03	0.16±0.05	G0	0.29
Nitrate reductase	NarG	K00370	nitrate reductase / nitrite oxidoreductase, alpha subunit	1.29±0.15	3.50±1.13	3.12±1.63	-1.27	0.17
Nitrate reductase	NapA	K02567	periplasmic nitrate reductase NapA	0.82±0.03	1.89±0.12	0.83±0.31	-0.02	1.20
Fumarate reductase	SdhA	K00239	succinate dehydrogenase / fumarate reductase, flavoprotein subunit	1842.42±170.02	685.06±92.14	605.90±61.17	1.60	0.18
Fumarate reductase	SdhB	K00240	succinate dehydrogenase / fumarate reductase, iron-sulfur subunit	953.78±86.22	516.18±33.16	480.20±74.78	0.99	0.10
Fumarate reductase	SdhC	K00241	succinate dehydrogenase /fumarate reductase, cytochrome b subunit	833.97±140.92	535.12±50.23	415.81±72.59	1.00	0.36

[1] mean value ± standard error; the unit of gene results is RPKM; the relative abundance of species results was normalized to 1,000,000 for each sample

[2] values are calculated form $log_2(G/B)$ or $log_2(P/B)$

KO: Kyoto Encyclopedia of Genes and Genomes orthology; EC: Enzyme Commission Number

Supplementary Table2. Genera composition of ruminal microbiome detected in this study

Domain	Genus	B	G	P
Archaea	Methanolobus	1497.32±365.86	2957.06±73.01	482.24±33.98
Archaea	Methanoplanus	633.62±235.74	3309.58±62.58	200.98±15.46
Archaea	Methanosaeta	612.09±86.43	804.06±22.76	412.59±21.82
Archaea	Methanobrevibacter	586.91±231.04	801.62±122.65	125.91±8.43
Archaea	Methanocella	492.72±166.02	1158.53±37.65	142.75±12.40
Archaea	Archaeoglobus	412.92±41.59	231.15±4.96	142.20±8.10
Archaea	Methanosarcina	403.75±134.08	869.96±70.36	104.57±4.85
Archaea	Thermococcus	376.65±68.98	599.36±5.71	172.47±9.20
Archaea	Methanococcus	357.87±104.97	3.17±1.12	838.26±87.91
Archaea	Methanosphaerula	314.17±58.97	271.00±15.19	681.16±31.62
Archaea	Haloquadratum	236.45±33.17	124.51±3.65	57.65±4.82
Archaea	Thermoplasma	205.66±45.82	505.92±9.74	114.81±5.62
Archaea	P_Euryarchaeota	171.95±15.51	265.58±3.39	71.87±3.64
Archaea	Methanosphaera	157.91±25.22	58.32±7.51	188.74±16.44
Archaea	Sulfolobus	154.17±21.20	229.34±23.80	31.13±1.36
Archaea	Methanococcoides	125.38±24.86	215.74±13.88	54.08±3.05
Archaea	Methanospirillum	100.68±29.86	208.33±11.12	25.58±2.04
Archaea	Methanoculleus	73.02±22.86	140.84±5.14	34.96±1.96
Archaea	Methanosalsum	67.67±5.86	64.54±3.81	21.33±2.09
Archaea	Pyrococcus	67.02±8.21	25.75±0.47	17.62±1.96
Archaea	Methanocorpusculum	65.17±20.91	152.97±0.97	37.96±3.26
Archaea	C_Methanomicrobia	61.41±12.09	142.28±3.14	44.73±2.29
Archaea	F_Methanosarcinaceae	61.26±5.25	53.50±1.98	24.90±2.09
Archaea	Methanohalophilus	57.53±23.27	71.26±4.49	14.49±0.77
Archaea	Halopiger	48.36±16.57	89.01±1.05	6.24±0.22
Archaea	Aciduliprofundum	44.77±6.48	18.18±1.41	7.87±1.13
Archaea	F_Halobacteriaceae	44.75±13.66	43.71±0.38	7.30±0.59
Archaea	Nitrosopumilus	42.07±16.39	36.40±3.77	15.45±0.28
Archaea	D_Archaea	41.99±7.41	14.08±0.75	15.76±1.40
Archaea	Methanobacterium	32.65±3.19	21.11±1.12	35.19±0.56
Archaea	Ferroglobus	30.71±6.05	12.75±0.62	8.38±0.90
Archaea	Ignisphaera	30.66±3.83	16.88±1.27	5.33±0.27
Archaea	Methanothermococcus	29.99±9.07	0.99±0.30	43.50±2.70
Archaea	F_Methanococcaceae	26.58±8.35	0.51±0.20	33.35±2.84

Archaea	Halogeometricum	25.78±2.47	33.14±2.74	14.12±1.08
Archaea	Candidatus_Korarchaeum	24.96±7.75	0	3.42±0.51
Archaea	Methanothermobacter	24.32±2.61	47.39±0.85	20.09±1.50
Archaea	Methanohalobium	22.34±3.23	58.08±6.38	15.40±0.81
Archaea	Metallosphaera	19.80±1.34	11.63±1.38	5.76±0.67
Archaea	Haloferax	19.08±7.92	56.40±2.33	2.13±0.15
Archaea	Methanocaldococcus	17.41±4.24	0.91±0.21	3.96±0.31
Archaea	Pyrobaculum	16.80±4.06	84.27±2.63	2.69±0.32
Archaea	Haloarcula	16.34±0.71	22.21±1.82	8.88±0.70
Archaea	Fervidicoccus	16.31±2.20	1.67±0.17	18.54±1.81
Archaea	F_Thermococcaceae	15.20±2.25	27.15±1.97	5.97±0.53
Archaea	O_Methanomicrobiales	14.71±3.32	55.70±2.40	12.75±0.71
Archaea	Methanopyrus	12.47±0.68	6.09±0.38	23.17±1.35
Archaea	Haloterrigena	10.62±3.94	23.06±0.89	0.56±0.12
Archaea	Methanotorris	10.43±1.95	3.69±0.47	20.75±1.41
Archaea	Acidianus	10.41±4.23	0.20±0.07	2.10±0.12
Archaea	Halorubrum	9.60±1.40	8.14±0.20	2.26±0.16
Archaea	Natrialba	8.84±2.76	2.60±0.48	2.96±0.09
Archaea	Methanoregula	8.24±3.77	18.52±1.32	0.11±0.04
Archaea	Cenarchaeum	6.44±2.76	9.38±0.38	1.23±0.14
Archaea	F_Methanobacteriaceae	5.94±1.82	9.75±1.55	0.97±0.19
Archaea	C_Thermoprotei	5.66±1.65	0.06±0.06	0.90±0.17
Archaea	Natrinema	4.59±2.38	1.23±0.29	0.89±0.14
Archaea	Natronomonas	4.30±1.85	4.44±0.63	1.00±0.15
Archaea	F_Sulfolobaceae	3.85±1.57	1.81±0.40	0.59±0.12
Archaea	Methanothermus	3.69±0.72	0.04±0.04	10.46±1.04
Archaea	Acidilobus	3.37±0.86	7.11±0.37	1.12±0.10
Archaea	Aeropyrum	3.14±0.96	0	0.21±0.06
Archaea	F_Archaeoglobaceae	3.00±0.76	1.37±0.42	0.84±0.22
Archaea	Nanoarchaeum	2.23±0.41	1.17±0.11	1.35±0.23
Archaea	Pyrolobus	1.63±0.49	1.23±0.39	0.28±0.06
Archaea	O_Methanosarcinales	1.26±0.15	0.75±0.33	2.45±0.23
Archaea	Thermogladius	1.25±0.33	3.19±0.72	0.07±0.02
Archaea	Halorhabdus	1.13±0.11	0.67±0.17	0.12±0.03
Archaea	Desulfurococcus	1.09±0.34	0	0.04±0.01
Archaea	Halalkalicoccus	0.82±0.31	0.43±0.03	0.04±0.02
Archaea	O_Methanococcales	0.77±0.25	0	1.00±0.15
Archaea	Thermoproteus	0.66±0.34	0.50±0.14	0

Archaea	Picrophilus	0.54±0.18	0	0.94±0.07
Archaea	Caldivirga	0.41±0.20	0.63±0.03	0
Archaea	Staphylothermus	0.33±0.11	0	2.07±0.09
Archaea	Halomicrobium	0.32±0.20	8.98±0.80	0
Archaea	Halobacterium	0.24±0.08	1.06±0.24	0.27±0.03
Archaea	F_Methanoregulaceae	0.20±0.10	0.67±0.17	0
Archaea	Thermofilum	0.02±0.02	1.16±0.11	0
Archaea	Thermosphaera	0.00±0.00	2.58±0.20	0
Archaea	Vulcanisaeta	0.00±0.00	0	0.67±0.10
Bacteria	D_Bacteria	248851.11±33787.66	116528.50±89.43	324250.50±1222.88
Bacteria	Prevotella	120980.10±5632.64	313562.25±2231.80	118130.37±10531.55
Bacteria	Clostridium	89575.26±7818.92	41821.55±2615.34	105568.22±10478.53
Bacteria	Bacteroides	30245.85±1741.11	48197.05±2245.70	22024.94±1401.77
Bacteria	Geobacter	26062.20±5116.75	31252.83±715.14	12041.04±874.03
Bacteria	Paenibacillus	22445.10±5588.99	37716.36±528.49	9027.91±579.77
Bacteria	P_Proteobacteria	19407.93±651.72	13430.62±464.40	16922.93±1115.72
Bacteria	Arcobacter	15405.71±4430.72	177.28±24.01	66951.28±8692.60
Bacteria	Bacillus	12390.04±2561.86	11204.64±602.55	3988.07±209.10
Bacteria	P_Firmicutes	12269.73±2275.33	8203.81±412.71	5242.89±274.11
Bacteria	Syntrophobacter	11972.29±1924.22	10009.41±436.68	5597.75±420.70
Bacteria	Eggerthella	11394.94±1740.74	14653.58±447.75	5238.02±298.85
Bacteria	Arthrobacter	11046.10±1660.36	10184.56±546.89	6978.63±527.38
Bacteria	Brachyspira	10613.96±3320.58	19.13±2.76	24636.70±2802.02
Bacteria	Desulfovibrio	8138.07±370.48	11547.80±468.17	6811.61±538.82
Bacteria	Granulicella	7947.49±249.14	20536.82±811.71	7048.57±569.68
Bacteria	O_Bacteroidales	7917.58±921.21	6384.48±198.04	7641.66±605.20
Bacteria	O_Clostridiales	6273.45±444.27	3742.20±211.22	7790.34±274.55
Bacteria	Marinobacter	6183.33±1797.18	4373.62±129.15	1607.70±122.94
Bacteria	Staphylococcus	6117.66±1291.61	1717.96±180.01	1433.13±26.59
Bacteria	Fibrobacter	5712.71±1233.72	1457.38±20.03	3552.48±204.56
Bacteria	Desulfomonile	5594.93±1564.64	5979.35±100.46	1729.81±101.43
Bacteria	Syntrophus	5442.42±1639.87	6988.11±127.80	1115.60±87.20
Bacteria	Akkermansia	5319.83±1273.47	10032.00±139.45	1834.23±155.04
Bacteria	Streptococcus	4889.88±1172.44	1176.75±77.33	1729.91±96.49
Bacteria	Sphaerochaeta	4732.17±180.54	4661.82±44.16	4775.82±370.73
Bacteria	Slackia	4563.37±162.41	19616.67±742.30	3451.33±268.65
Bacteria	Sinorhizobium	4511.90±284.38	4128.77±157.02	1989.36±111.67
Bacteria	O_Rhizobiales	4461.32±204.87	3024.63±99.66	2527.94±167.89

Bacteria	C_Deltaproteobacteria	4247.48±362.81	3950.94±74.04	3039.03±228.06
Bacteria	Oscillibacter	4223.89±979.33	6444.59±81.96	1787.72±152.19
Bacteria	Spirochaeta	4211.80±219.82	5793.78±112.46	1695.30±116.81
Bacteria	Selenomonas	4162.83±441.55	4306.76±47.96	2423.84±216.33
Bacteria	Bifidobacterium	4148.85±437.79	4609.74±212.05	3548.70±277.91
Bacteria	Pelobacter	4050.84±1327.18	3623.58±100.11	853.39±65.60
Bacteria	P_Bacteroidetes	4014.60±405.60	2068.98±112.13	3784.29±209.64
Bacteria	C_Gammaproteobacteria	3972.81±155.66	2300.09±26.37	3325.83±248.79
Bacteria	Butyrivibrio	3798.28±769.89	6624.97±438.10	2189.57±198.73
Bacteria	Marivirga	3526.72±1485.95	656.12±62.77	413.63±14.87
Bacteria	Borrelia	3408.18±1057.00	36.90±3.26	6053.56±641.34
Bacteria	Candidatus_Phytoplasma	3271.25±1031.53	21.20±1.78	14611.13±1923.01
Bacteria	Robiginitalea	3264.48±1148.05	853.92±19.20	565.64±48.75
Bacteria	Flavobacterium	3186.81±452.39	514.88±61.67	4508.37±273.35
Bacteria	Terriglobus	3164.50±104.27	4005.43±138.73	2345.74±173.43
Bacteria	O_Bacillales	3011.18±524.13	1491.78±70.55	1164.44±68.02
Bacteria	Lactobacillus	3007.55±393.92	961.98±86.94	1336.63±65.81
Bacteria	Desulfomicrobium	2912.44±766.89	4119.75±99.03	1006.58±68.50
Bacteria	C_Actinobacteria	2832.69±398.05	2414.39±124.94	2668.26±208.75
Bacteria	Azospira	2613.87±816.50	3378.95±142.42	568.62±43.57
Bacteria	O_Actinomycetales	2597.49±594.02	735.17±38.28	3269.55±272.11
Bacteria	Candidatus_Koribacter	2594.46±184.43	1603.32±52.28	957.32±47.73
Bacteria	Desulfatibacillum	2550.46±645.37	2188.32±19.78	736.27±61.91
Bacteria	Brevibacillus	2517.20±332.15	2845.16±77.04	1574.19±99.29
Bacteria	Wigglesworthia	2467.57±777.53	0.88±0.18	9489.28±1211.76
Bacteria	C_Bacilli	2467.05±186.25	525.97±40.80	1093.11±45.07
Bacteria	Methylococcus	2462.35±358.14	1989.53±109.76	1459.98±121.20
Bacteria	C_Alphaproteobacteria	2443.51±288.50	1251.09±60.31	2115.46±166.63
Bacteria	Treponema	2351.70±119.14	1428.54±45.06	2506.19±218.49
Bacteria	Ruminococcus	2327.87±71.85	4814.41±217.66	903.60±100.84
Bacteria	Tannerella	2288.46±259.11	1474.95±32.22	2782.66±132.86
Bacteria	Pseudovibrio	2272.25±153.28	2975.07±44.64	2291.87±192.10
Bacteria	Vibrio	2192.00±210.74	1776.62±25.31	2134.41±123.82
Bacteria	Alcanivorax	2154.37±819.75	919.55±54.40	256.92±21.87
Bacteria	Marinitoga	2139.87±597.51	176.38±21.93	3280.50±293.18
Bacteria	Coraliomargarita	2073.72±193.41	2337.13±51.46	2469.55±152.27
Bacteria	Leptotrichia	2018.10±600.67	39.84±5.22	3607.74±319.43
Bacteria	Ethanoligenens	1926.26±343.19	1723.53±11.56	628.80±51.73

Bacteria	F_Acidobacteriaceae	1907.58±201.31	2303.07±98.39	1957.27±162.37
Bacteria	Aliivibrio	1901.81±590.59	514.69±41.13	224.63±7.82
Bacteria	Desulfosporosinus	1876.97±291.68	3005.00±201.28	1061.63±45.45
Bacteria	Roseburia	1853.81±592.51	4528.72±128.97	433.11±36.99
Bacteria	Mycoplasma	1847.59±505.33	85.32±10.40	6766.01±756.24
Bacteria	Burkholderia	1843.42±66.91	911.55±47.53	1120.93±60.30
Bacteria	Corynebacterium	1796.13±124.83	1437.92±41.41	1670.90±123.93
Bacteria	Olsenella	1781.10±251.13	7613.55±349.95	1102.43±87.66
Bacteria	Eubacterium	1779.65±237.41	1711.97±99.36	1137.93±101.82
Bacteria	Candidatus_Solibacter	1767.29±60.92	1334.79±54.62	774.89±33.88
Bacteria	Alistipes	1737.19±45.26	2538.77±26.44	1316.72±106.82
Bacteria	Hyphomicrobium	1731.12±256.96	2723.45±56.66	777.95±44.22
Bacteria	Fusobacterium	1665.28±505.92	2.94±0.21	5077.73±574.06
Bacteria	Sorangium	1644.75±59.63	1546.58±83.19	863.13±42.99
Bacteria	Rhodomicrobium	1631.82±79.04	807.00±29.05	665.82±22.29
Bacteria	Thermotoga	1626.78±211.38	556.27±39.02	653.24±56.87
Bacteria	Oceanobacillus	1614.80±450.99	626.78±58.22	297.47±5.42
Bacteria	Opitutus	1591.33±61.82	815.78±43.23	715.46±50.57
Bacteria	Pelodictyon	1518.58±199.12	1399.52±21.84	1116.97±87.97
Bacteria	Desulfitobacterium	1506.77±398.41	1807.64±69.75	546.22±45.35
Bacteria	Chlorobium	1429.78±317.53	837.90±9.63	711.29±49.60
Bacteria	Chitinophaga	1403.40±198.26	1551.81±70.46	763.89±59.45
Bacteria	C_Clostridia	1374.48±80.33	791.80±76.43	1058.83±40.92
Bacteria	Agrobacterium	1355.04±270.50	1548.55±49.92	436.18±29.52
Bacteria	Fluviicola	1350.07±235.87	414.74±38.48	590.00±15.32
Bacteria	Pseudomonas	1331.79±174.14	3381.60±158.02	1503.39±121.94
Bacteria	Bradyrhizobium	1311.30±50.45	1321.52±70.15	643.83±32.72
Bacteria	Desulfotomaculum	1292.42±242.01	1095.93±23.68	487.15±37.12
Bacteria	Melioribacter	1231.43±119.38	616.26±35.79	1010.29±115.21
Bacteria	Alicyclobacillus	1210.98±243.76	592.33±15.31	367.29±20.63
Bacteria	O_Desulfuromonadales	1206.64±296.07	1139.53±16.32	433.17±34.32
Bacteria	Finegoldia	1204.92±276.16	333.69±36.83	519.20±15.11
Bacteria	Dehalobacter	1189.34±524.46	1318.63±75.82	99.31±6.54
Bacteria	O_Lactobacillales	1157.63±136.05	141.46±11.41	368.97±14.68
Bacteria	Azospirillum	1115.30±56.65	665.76±29.04	528.46±42.84
Bacteria	Psychroflexus	1115.09±365.00	275.27±30.31	109.77±2.72
Bacteria	Candidatus_Tremblaya	1094.45±251.92	151.85±1.59	1220.59±74.40
Bacteria	Sphingobacterium	1083.41±126.33	1245.86±91.90	683.60±26.03

Bacteria	Acidovorax	1077.67±194.99	5209.02±258.20	434.55±35.48
Bacteria	Bacteriovorax	1055.46±107.72	268.15±25.39	407.58±8.97
Bacteria	Chloroherpeton	1048.77±18.14	683.46±18.21	983.42±88.25
Bacteria	F_Peptostreptococcaceae	1030.13±105.90	362.88±45.13	1618.53±90.22
Bacteria	Geobacillus	1025.78±120.41	1021.46±34.85	547.60±53.14
Bacteria	Acidithiobacillus	999.87±220.71	670.02±37.91	537.20±39.94
Bacteria	Chelativorans	997.61±165.34	934.99±30.46	284.33±16.57
Bacteria	Maribacter	994.63±136.79	1821.46±170.66	646.40±23.28
Bacteria	Propionibacterium	987.77±134.06	1470.30±73.53	1276.68±108.14
Bacteria	Desulfurispirillum	954.41±95.89	733.20±23.48	597.36±51.04
Bacteria	Porphyromonas	944.38±209.71	275.49±7.22	1099.53±75.44
Bacteria	Methylobacterium	939.58±87.37	1396.50±27.07	509.81±35.90
Bacteria	Prochlorococcus	926.80±50.18	249.05±28.21	796.41±32.42
Bacteria	Mesotoga	915.68±67.34	1352.02±37.16	489.84±36.67
Bacteria	Shewanella	904.54±156.91	1314.29±3.61	1505.98±99.40
Bacteria	Ochrobactrum	896.57±57.01	933.87±9.44	426.85±27.17
Bacteria	Chlorobaculum	881.60±60.84	1263.60±33.80	694.63±48.29
Bacteria	F_Bacillaceae	877.24±134.51	340.54±29.12	401.35±25.83
Bacteria	Stigmatella	874.14±34.45	624.83±29.68	770.28±66.22
Bacteria	Enterococcus	867.02±81.90	147.11±14.97	200.98±4.34
Bacteria	Cellulosilyticum	849.37±308.28	354.17±34.85	172.94±11.59
Bacteria	Acidaminococcus	840.34±190.21	842.79±11.46	353.67±21.93
Bacteria	F_Enterobacteriaceae	834.84±141.07	169.31±5.62	979.77±51.05
Bacteria	Symbiobacterium	830.46±274.39	686.68±38.97	543.91±62.86
Bacteria	Rhodococcus	822.20±96.65	337.12±19.40	652.30±44.40
Bacteria	Planctomyces	815.55±126.89	627.85±13.96	808.35±81.59
Bacteria	Thermoanaerobacterium	794.49±172.62	424.53±43.14	381.98±12.17
Bacteria	Prosthecochloris	789.87±237.17	1118.53±13.34	262.67±21.79
Bacteria	P_Acidobacteria	780.38±61.68	397.58±14.86	564.94±41.53
Bacteria	F_Rhizobiaceae	750.00±63.75	575.29±27.79	412.45±37.06
Bacteria	Ignavibacterium	742.40±101.49	158.13±15.73	552.01±29.55
Bacteria	Flexibacter	741.40±204.22	54.92±6.32	1371.22±96.86
Bacteria	C_Betaproteobacteria	730.28±39.26	1456.93±60.48	597.11±43.64
Bacteria	Xanthobacter	720.07±41.49	732.73±36.45	432.97±33.33
Bacteria	O_Myxococcales	664.80±47.30	137.43±4.42	586.93±38.14
Bacteria	Parvibaculum	659.05±197.32	943.29±24.26	150.63±9.54
Bacteria	Listeria	650.79±132.74	110.85±13.29	164.41±7.58
Bacteria	Belliella	629.61±150.38	168.78±13.85	261.62±12.81

Bacteria	Alkaliphilus	603.25±141.68	365.59±36.15	274.49±13.22
Bacteria	Mycobacterium	602.64±27.74	629.17±27.78	515.17±41.47
Bacteria	Cellulophaga	601.16±186.70	114.74±13.16	106.86±4.38
Bacteria	Sideroxydans	596.09±104.30	639.13±25.59	566.84±40.05
Bacteria	Chloroflexus	591.30±129.87	91.23±5.56	559.51±32.03
Bacteria	F_Clostridiaceae	583.08±92.94	202.73±22.56	324.18±15.53
Bacteria	Pedobacter	579.66±77.83	667.70±62.05	237.67±15.87
Bacteria	Streptomyces	569.38±20.59	232.50±13.05	479.27±40.23
Bacteria	Paludibacter	561.30±28.16	750.92±58.95	474.80±20.85
Bacteria	Beijerinckia	558.80±104.05	571.23±16.96	256.40±18.15
Bacteria	F_Paenibacillaceae	544.53±38.27	393.22±11.17	341.85±24.67
Bacteria	F_Coriobacteriaceae	544.15±56.25	1128.37±34.86	472.71±33.40
Bacteria	Runella	534.33±69.90	345.11±10.55	676.45±41.04
Bacteria	F_Peptococcaceae	506.30±100.76	538.81±30.57	266.53±18.07
Bacteria	Dyadobacter	489.75±53.41	236.85±4.23	369.45±24.35
Bacteria	Denitrovibrio	486.71±36.83	325.95±18.84	344.74±22.93
Bacteria	Marinomonas	483.01±26.08	826.86±49.91	379.56±21.40
Bacteria	Teredinibacter	482.51±34.85	408.31±11.93	373.24±16.99
Bacteria	Aeromonas	458.48±49.96	1535.92±43.26	655.11±57.25
Bacteria	Filifactor	457.00±35.19	308.44±27.94	216.78±13.72
Bacteria	Sulfurovum	455.41±77.38	510.88±9.58	201.67±16.99
Bacteria	Thioalkalivibrio	438.05±161.02	459.33±30.03	119.30±12.04
Bacteria	Nitrospira	436.56±26.21	431.40±11.16	301.49±23.02
Bacteria	Parabacteroides	434.75±75.15	115.25±5.14	529.27±28.85
Bacteria	Deinococcus	433.33±46.74	1185.55±71.68	334.14±31.66
Bacteria	Leuconostoc	426.86±58.76	25.65±1.59	107.04±5.54
Bacteria	Leptospira	417.51±96.79	29.14±3.92	387.07±4.02
Bacteria	Haliscomenobacter	415.93±59.84	438.03±1.12	606.39±35.96
Bacteria	F_Flavobacteriaceae	415.72±82.12	163.27±18.00	393.11±18.30
Bacteria	F_Chlorobiaceae	415.10±54.73	502.80±7.75	282.00±23.26
Bacteria	Syntrophobotulus	406.10±159.56	464.64±29.37	51.15±3.88
Bacteria	Nitrosospira	400.17±69.36	672.48±2.97	172.63±12.44
Bacteria	Anaeromyxobacter	386.95±23.39	92.33±5.17	250.12±17.74
Bacteria	Caldicellulosiruptor	385.51±46.79	253.44±25.72	357.99±24.84
Bacteria	Azorhizobium	378.47±12.96	430.26±20.44	302.98±24.97
Bacteria	O_Burkholderiales	377.39±15.19	445.94±32.78	344.75±26.35
Bacteria	Exiguobacterium	374.14±27.10	173.22±5.65	367.20±27.45
Bacteria	Neisseria	372.38±16.39	33.52±0.04	325.19±31.00

Bacteria	Anaerobaculum	371.87±101.96	426.90±6.93	172.53±12.25
Bacteria	Fervidobacterium	368.21±24.31	259.81±21.53	250.60±17.90
Bacteria	Comamonas	364.98±20.42	1267.56±34.00	426.17±37.35
Bacteria	F_Xanthobacteraceae	361.26±40.49	458.92±20.97	338.10±27.55
Bacteria	Methylobacillus	359.94±15.52	427.70±16.37	981.15±59.48
Bacteria	Legionella	356.80±18.20	1030.79±81.29	412.62±14.66
Bacteria	Jonesia	354.92±41.79	33.69±2.11	266.88±24.10
Bacteria	Salinibacter	352.00±10.91	317.96±13.79	306.71±22.03
Bacteria	Microbacterium	349.87±25.65	202.79±9.11	129.29±6.39
Bacteria	F_Syntrophaceae	343.97±115.53	364.78±4.73	75.40±5.86
Bacteria	Ehrlichia	338.14±57.90	77.55±7.51	473.31±23.72
Bacteria	Campylobacter	338.08±45.24	20.26±3.10	470.64±40.81
Bacteria	Nitrosococcus	332.84±69.04	250.75±5.86	180.68±11.91
Bacteria	Glaciecola	326.13±64.55	319.28±19.40	156.60±8.47
Bacteria	Lysinibacillus	323.27±54.04	599.10±52.74	228.01±7.47
Bacteria	O_Spirochaetales	319.77±100.30	0	452.96±44.43
Bacteria	P_Alphaproteobacteria	312.92±30.09	301.24±17.92	407.95±23.33
Bacteria	Achromobacter	305.69±108.53	360.02±29.10	64.70±6.57
Bacteria	Hahella	297.63±15.27	454.34±3.02	418.70±39.04
Bacteria	Rhizobium	295.19±102.25	172.78±8.01	53.42±4.61
Bacteria	Variovorax	292.32±37.59	404.17±15.62	244.97±15.92
Bacteria	Hirschia	285.48±40.16	134.68±6.06	234.07±12.66
Bacteria	Zobellia	284.51±30.31	250.21±12.80	147.82±10.41
Bacteria	O_Desulfovibrionales	281.93±39.45	290.63±11.99	151.17±11.77
Bacteria	Synechococcus	281.58±64.80	70.90±2.61	321.34±28.38
Bacteria	Rhodospirillum	281.22±7.80	170.55±12.67	254.05±22.42
Bacteria	O_Cytophagales	280.20±60.28	21.87±1.83	120.21±5.23
Bacteria	Desulfococcus	275.97±70.86	345.81±4.61	102.04±7.85
Bacteria	Segniliparus	275.33±10.10	141.36±5.99	151.17±15.03
Bacteria	Halanaerobium	274.00±80.04	43.10±5.87	567.02±38.69
Bacteria	Xylella	272.56±22.24	362.74±5.33	279.46±19.67
Bacteria	Desulfotalea	271.73±6.84	394.89±16.39	208.12±17.20
Bacteria	Elusimicrobium	271.44±17.06	77.05±5.99	235.18±37.87
Bacteria	Carnobacterium	264.56±72.85	47.38±2.63	25.51±0.89
Bacteria	Sebaldella	263.64±58.10	23.96±4.23	129.30±12.41
Bacteria	P_Gammaproteobacteria	263.32±36.73	207.84±3.08	254.34±12.34
Bacteria	Helicobacter	260.68±65.42	4.77±0.47	294.05±13.28
Bacteria	Melissococcus	260.38±71.60	41.04±5.39	96.94±4.58

Bacteria	F_Elusimicrobia	259.43±20.26	79.84±6.81	165.91±11.13
Bacteria	Kosmotoga	258.94±74.32	38.50±2.77	79.96±5.65
Bacteria	F_Comamonadaceae	256.40±11.45	1021.63±49.87	268.56±25.76
Bacteria	Atopobium	252.98±40.00	262.81±8.16	273.05±18.46
Bacteria	C_Mollicutes	248.23±77.20	0.97±0.17	1169.63±138.39
Bacteria	Corallococcus	243.88±23.67	147.45±9.65	299.62±22.92
Bacteria	Thermocrinis	241.56±74.93	2.74±0.26	76.94±7.33
Bacteria	Methylocella	240.74±11.84	332.23±7.99	134.17±12.99
Bacteria	Phenylobacterium	237.97±12.99	670.95±38.60	211.83±22.27
Bacteria	Pectobacterium	236.73±35.42	322.10±2.22	264.57±16.48
Bacteria	Leptospirillum	233.25±8.04	146.06±5.36	206.46±17.01
Bacteria	Rothia	229.31±52.32	132.38±3.03	266.92±19.58
Bacteria	Acaryochloris	227.30±4.40	485.35±14.68	240.91±16.57
Bacteria	O_Fusobacteriales	223.08±69.47	0.05±0.05	610.86±61.40
Bacteria	Acidobacterium	221.00±6.71	251.71±7.66	150.32±12.46
Bacteria	Thauera	218.35±17.40	502.03±29.32	98.22±6.69
Bacteria	Lactococcus	215.56±27.63	80.93±8.87	96.00±2.20
Bacteria	Solitalea	215.03±8.26	288.46±23.71	130.50±9.12
Bacteria	Gluconobacter	214.72±43.92	384.73±18.50	109.04±11.00
Bacteria	Desulfobulbus	214.34±19.41	161.22±4.60	146.29±11.48
Bacteria	F_Thermotogaceae	213.79±35.63	121.87±3.70	158.95±9.35
Bacteria	Cytophaga	213.39±11.34	55.57±4.86	116.47±10.20
Bacteria	Emticicia	211.44±35.28	114.81±10.45	184.46±8.84
Bacteria	Magnetospirillum	211.27±51.13	146.35±10.82	69.74±6.57
Bacteria	Halomonas	209.69±20.91	565.85±33.74	133.06±12.17
Bacteria	Heliobacterium	207.51±8.47	74.06±1.31	173.12±11.25
Bacteria	Muricauda	204.46±26.65	142.98±6.18	301.65±11.01
Bacteria	F_Flavobacteriaceae	204.43±11.59	34.64±1.99	147.50±9.27
Bacteria	Erysipelothrix	204.02±57.43	11.02±1.12	43.02±2.48
Bacteria	Candidatus Desulforudis	201.40±49.44	89.83±2.60	96.53±6.99
Bacteria	Thermodesulfobacterium	196.45±60.13	5.10±0.73	398.13±29.86
Bacteria	Pusillimonas	191.00±6.84	367.45±22.84	247.45±19.29
Bacteria	Macrococcus	190.28±6.11	504.39±43.17	99.78±7.80
Bacteria	Spirosoma	189.24±37.75	220.34±2.97	252.52±18.34
Bacteria	O_Flavobacteriales	187.59±9.17	257.52±33.35	144.92±5.13
Bacteria	Bdellovibrio	186.40±12.44	219.45±5.44	143.45±10.92
Bacteria	Thermobaculum	186.00±38.50	81.00±0.69	224.23±19.12
Bacteria	Ralstonia	184.03±11.26	465.08±25.47	119.42±8.33

Bacteria	Novosphingobium	182.92±6.83	53.52±4.51	126.36±9.02
Bacteria	Thermus	182.76±53.82	6.61±0.77	113.38±5.04
Bacteria	Herbaspirillum	182.64±29.03	132.11±4.30	94.45±9.10
Bacteria	Aminobacterium	182.14±46.40	128.36±10.33	65.73±4.33
Bacteria	Odoribacter	180.68±44.74	52.18±3.47	50.08±4.37
Bacteria	Granulibacter	177.23±53.92	238.82±10.61	32.26±3.68
Bacteria	Capnocytophaga	176.83±11.18	36.53±4.33	152.97±12.01
Bacteria	Parachlamydia	175.26±9.00	93.27±6.46	153.64±5.74
Bacteria	Rhodopirellula	170.28±38.59	66.04±3.94	190.38±17.21
Bacteria	F_Porphyromonadaceae	170.02±44.49	15.15±0.30	215.77±10.96
Bacteria	Candidatus Methylomirabilis	169.50±19.53	239.16±12.44	179.53±12.45
Bacteria	F_Myxococcaceae	168.29±42.55	16.34±1.24	241.17±19.37
Bacteria	Caulobacter	165.24±32.95	88.49±4.76	197.60±17.58
Bacteria	Syntrophothermus	157.89±24.32	74.21±2.44	68.85±5.42
Bacteria	Catenulispora	157.73±10.42	177.71±13.37	112.55±9.77
Bacteria	Blattabacterium	157.52±35.41	60.33±3.50	164.29±12.44
Bacteria	Sulfurimonas	157.08±30.86	13.81±1.47	186.75±5.12
Bacteria	Aromatoleum	156.45±18.62	307.82±13.00	81.16±5.03
Bacteria	Myxococcus	154.65±27.33	223.52±15.79	217.01±16.95
Bacteria	Enterobacter	152.64±26.38	247.08±6.01	164.63±12.46
Bacteria	Thermacetogenium	151.34±10.88	144.00±3.73	83.32±6.61
Bacteria	Nitrosomonas	151.10±9.57	109.01±3.59	108.83±7.04
Bacteria	Dechloromonas	150.72±13.86	92.63±4.01	122.46±9.79
Bacteria	Leadbetterella	147.63±19.55	35.50±3.76	91.07±5.60
Bacteria	Parvularcula	147.51±20.79	74.02±1.55	84.85±6.53
Bacteria	Alteromonas	146.49±22.31	73.31±5.45	151.74±10.56
Bacteria	Polaromonas	144.93±9.20	486.71±13.80	212.10±18.00
Bacteria	Dickeya	144.24±7.80	47.56±2.24	165.54±10.68
Bacteria	Cyanothece	143.90±33.17	12.12±0.43	112.62±6.29
Bacteria	Simkania	143.36±4.07	164.07±13.34	118.52±7.91
Bacteria	Starkeya	139.70±11.58	23.68±0.87	97.69±8.07
Bacteria	O_Alteromonadales	139.01±25.38	86.54±2.53	160.98±12.84
Bacteria	Aequorivita	138.84±27.16	16.10±1.85	103.58±5.86
Bacteria	F_Hyphomicrobiaceae	138.78±28.12	54.25±3.09	98.94±3.88
Bacteria	Methylomonas	135.49±12.74	55.47±0.26	67.26±3.94
Bacteria	O_Syntrophobacterales	135.24±18.24	66.60±1.09	58.92±6.61
Bacteria	Deferribacter	134.14±27.22	31.87±3.77	117.56±7.09

Bacteria	Rubrobacter	133.71±23.83	41.11±2.07	70.15±7.10
Bacteria	Acetobacterium	130.10±20.50	18.51±1.73	107.59±4.48
Bacteria	Ammonifex	128.42±10.60	32.58±0.20	88.02±6.20
Bacteria	Megasphaera	123.36±18.99	55.71±1.87	164.02±13.30
Bacteria	Francisella	122.45±16.79	83.94±9.95	128.94±5.73
Bacteria	Acinetobacter	121.27±19.66	53.03±4.34	95.24±5.96
Bacteria	Serratia	120.58±29.49	21.48±0.15	396.12±57.48
Bacteria	Escherichia	119.71±14.28	31.49±4.04	135.48±8.23
Bacteria	Gallionella	117.75±16.59	9.21±0.64	109.95±6.49
Bacteria	Candidatus Carsonella	117.09±22.45	229.49±6.80	150.30±11.26
Bacteria	Thermosipho	113.95±7.66	24.30±2.82	107.50±5.02
Bacteria	Petrotoga	112.94±5.96	40.53±4.91	59.63±2.16
Bacteria	Cupriavidus	111.87±6.69	97.02±9.12	95.24±7.56
Bacteria	Alicycliphilus	111.52±10.16	2329.86±81.72	77.38±6.34
Bacteria	Cryptobacterium	110.87±29.15	34.90±0.38	120.97±7.37
Bacteria	Desulfobacula	110.04±6.12	26.14±1.74	74.17±6.36
Bacteria	Mahella	109.74±23.00	120.88±11.08	113.62±11.01
Bacteria	Conexibacter	108.01±28.98	3.77±0.50	82.79±6.07
Bacteria	Dictyoglomus	106.69±20.01	22.88±1.60	74.07±3.91
Bacteria	Amycolicicoccus	106.07±5.21	75.04±3.67	69.80±5.09
Bacteria	Sulfuricurvum	106.00±7.90	25.04±1.69	91.90±5.84
Bacteria	Xenorhabdus	105.38±6.63	34.25±2.09	91.67±7.43
Bacteria	Methylomicrobium	104.77±18.21	53.33±4.05	52.22±5.66
Bacteria	Bartonella	103.64±8.77	37.66±3.95	134.44±4.30
Bacteria	F_Spirochaetaceae	103.50±2.27	39.69±2.02	92.73±7.85
Bacteria	F_Nostocaceae	102.48±29.33	70.19±7.41	107.51±6.92
Bacteria	F_Burkholderiaceae	102.44±16.09	85.37±4.21	96.75±5.87
Bacteria	Hyphomonas	101.78±25.97	112.17±8.39	28.08±2.66
Bacteria	F_Rhodospirillaceae	101.39±2.84	51.18±1.25	95.52±7.51
Bacteria	Ramlibacter	100.98±37.52	125.22±9.22	34.08±3.09
Bacteria	C_Cyanobacteria	100.54±21.33	3.29±0.53	121.12±7.93
Bacteria	Turneriella	100.50±25.01	15.10±1.29	112.01±9.69
Bacteria	F_Pasteurellaceae	100.27±22.26	10.96±0.96	201.56±21.66
Bacteria	Pelotomaculum	99.44±13.03	25.28±0.54	35.37±3.47
Bacteria	Advenella	98.43±11.85	42.82±1.54	74.52±5.28
Bacteria	Nostoc	96.68±4.75	235.21±15.54	76.09±4.42
Bacteria	Cyclobacterium	96.37±18.30	88.42±9.90	121.64±8.57
Bacteria	Caldilinea	96.32±8.69	117.71±2.61	92.77±8.27

Bacteria	Chlamydia	95.81±26.29	7.49±1.56	59.40±4.31
Bacteria	Gluconacetobacter	95.74±19.03	36.67±2.53	97.32±8.06
Bacteria	Mobiluncus	95.16±28.12	0	72.28±8.18
Bacteria	Actinobacillus	94.40±9.55	89.39±6.46	119.70±14.66
Bacteria	Gemmatimonas	94.38±20.76	27.92±2.10	134.67±11.47
Bacteria	Trichodesmium	93.94±25.38	7.38±0.74	114.56±4.79
Bacteria	Sphingobium	92.49±1.90	48.16±2.93	67.78±6.08
Bacteria	Kyrpidia	90.68±12.65	33.70±2.18	90.49±4.76
Bacteria	Halothiobacillus	90.23±12.60	110.39±1.70	101.41±7.37
Bacteria	Sulfobacillus	90.14±13.89	12.88±0.40	93.58±4.95
Bacteria	Methylacidiphilum	89.13±7.16	75.04±1.19	73.72±4.80
Bacteria	Aggregatibacter	88.37±4.80	34.21±0.79	188.23±17.93
Bacteria	Halobacillus	88.26±5.19	354.11±22.91	34.16±2.51
Bacteria	Candidatus Chloracidobacterium	87.73±24.85	5.29±0.59	138.17±11.75
Bacteria	Thermanaerovibrio	87.55±13.12	7.83±0.89	103.29±7.39
Bacteria	Colwellia	86.28±10.66	137.82±13.17	56.87±3.43
Bacteria	Streptobacillus	85.24±26.45	0	283.11±29.06
Bacteria	Thiobacillus	85.13±13.08	48.45±3.28	59.41±2.42
Bacteria	Caldanaerobacter	84.74±20.87	1.18±0.25	12.83±1.32
Bacteria	Acholeplasma	84.27±14.67	89.62±8.76	68.03±2.16
Bacteria	Nitratifractor	82.83±5.78	12.48±0.11	87.32±6.42
Bacteria	Leifsonia	82.51±4.36	43.24±1.93	39.11±3.37
Bacteria	Albidiferax	81.91±13.57	97.12±4.83	172.86±11.33
Bacteria	Nocardiopsis	81.12±9.01	81.78±3.84	82.98±6.99
Bacteria	O_Clostridiales	81.03±13.70	67.42±4.61	47.83±3.05
Bacteria	Nautilia	80.17±19.86	4.66±0.67	96.76±6.57
Bacteria	Candidatus Arthromitus	78.98±15.79	56.86±8.92	119.34±5.73
Bacteria	F_Desulfobacteraceae	78.48±18.98	12.72±0.82	28.92±3.01
Bacteria	Coprothermobacter	76.20±15.26	6.95±0.25	38.65±3.52
Bacteria	Amycolatopsis	75.47±12.92	16.04±1.39	53.21±3.87
Bacteria	Desulfohalobium	75.07±8.69	82.25±5.38	63.83±3.70
Bacteria	Salmonella	73.87±10.38	62.23±0.54	62.42±6.40
Bacteria	Candidatus Liberibacter	72.71±14.81	19.76±1.32	37.41±1.80
Bacteria	F_Cryomorphaceae	72.59±6.83	64.63±4.36	67.82±5.13
Bacteria	Methylibium	72.51±22.81	273.01±16.18	23.78±2.69
Bacteria	Pseudogulbenkiania	72.46±3.03	844.43±14.20	47.79±3.80
Bacteria	Zymomonas	71.16±4.22	23.65±0.65	45.12±3.88

Bacteria	Zymomonas	71.16±4.22	23.65±0.65	45.12±3.88
Bacteria	Roseobacter	69.25±12.84	46.50±1.21	60.28±5.03
Bacteria	Gardnerella	69.25±11.30	46.77±3.67	82.50±6.15
Bacteria	Veillonella	68.24±14.85	28.23±1.94	59.16±3.88
Bacteria	Ornithobacterium	68.00±14.92	3.47±0.69	46.85±3.05
Bacteria	Buchnera	67.79±16.37	11.84±1.73	185.50±14.53
Bacteria	Rickettsia	66.80±5.82	1.74±0.28	55.50±2.29
Bacteria	Desulfurobacterium	66.07±17.49	2.26±0.53	131.23±9.73
Bacteria	Coriobacterium	65.59±6.57	441.44±15.23	36.85±3.51
Bacteria	Riemerella	65.50±6.45	134.10±14.91	39.80±2.07
Bacteria	O_Desulfobacterales	64.40±20.00	5.00±0.15	18.65±1.84
Bacteria	F_Bradyrhizobiaceae	61.33±16.04	24.59±1.98	41.18±3.15
Bacteria	Zunongwangia	61.30±6.42	12.78±2.47	33.80±1.55
Bacteria	Tepidanaerobacter	61.23±4.84	40.40±5.17	45.43±2.42
Bacteria	Klebsiella	60.45±11.07	133.82±5.20	27.38±2.90
Bacteria	Intrasporangium	60.33±14.58	141.64±6.09	16.90±0.66
Bacteria	Rahnella	60.25±16.76	123.40±2.68	24.67±1.76
Bacteria	Dinoroseobacter	59.77±20.75	10.92±1.87	13.73±1.91
Bacteria	Desulfobacca	58.97±2.73	59.00±2.13	32.86±2.37
Bacteria	Rhodobacter	58.59±1.82	19.61±0.75	48.97±3.40
Bacteria	Moorella	57.99±5.05	2.59±0.11	18.56±0.77
Bacteria	Weeksella	57.67±10.19	6.24±0.44	75.59±2.04
Bacteria	Niastella	56.54±3.14	14.80±0.53	49.55±2.81
Bacteria	Oenococcus	56.25±13.39	5.30±0.98	42.21±1.76
Bacteria	Alkalilimnicola	54.74±8.19	107.55±4.80	22.73±2.70
Bacteria	Thermincola	54.34±12.44	8.76±0.82	19.73±1.63
Bacteria	Stackebrandtia	54.11±14.52	1.62±0.16	98.66±7.04
Bacteria	F_Phyllobacteriaceae	53.76±12.87	95.86±4.22	14.91±1.27
Bacteria	Wolinella	53.29±14.40	3.83±0.51	73.93±7.98
Bacteria	Streptosporangium	53.26±5.96	85.64±6.50	47.14±5.62
Bacteria	Yersinia	53.23±9.50	25.87±0.78	84.58±5.72
Bacteria	O_Selenomonadales	52.44±7.70	43.19±1.15	54.86±4.92
Bacteria	Desulfobacterium	51.85±7.13	12.96±0.52	69.78±5.05
Bacteria	Basfia	51.41±6.93	6.82±0.20	40.05±5.08
Bacteria	Clavibacter	50.92±4.23	58.71±3.73	36.50±1.74
Bacteria	Methylovorus	50.50±6.14	34.89±1.63	83.26±7.26
Bacteria	Coxiella	50.42±3.66	14.30±0.63	61.87±3.72
Bacteria	Dehalococcoides	48.87±14.57	1.88±0.45	36.83±2.97

Bacteria	Chromobacterium	48.73±9.79	28.55±1.76	30.30±2.82
Bacteria	Saccharomonospora	48.28±1.22	14.16±1.17	49.15±4.99
Bacteria	Candidatus Zinderia	48.17±11.97	0.91±0.20	98.71±9.94
Bacteria	Mesorhizobium	47.89±9.19	10.22±0.71	81.19±5.12
Bacteria	Rhodothermus	47.58±1.44	217.48±6.65	37.52±3.73
Bacteria	O_Thermoanaerobacterales	47.49±7.49	30.86±2.08	48.96±4.16
Bacteria	Candidatus Azobacteroides	47.34±5.00	102.26±11.09	22.89±1.18
Bacteria	Pirellula	47.31±12.34	14.33±0.28	81.40±5.60
Bacteria	F_Sphingobacteriaceae	46.80±6.02	42.68±5.23	31.47±2.06
Bacteria	Janthinobacterium	46.62±11.74	5.71±0.30	39.28±3.18
Bacteria	F_Caulobacteraceae	46.16±6.71	53.47±4.65	58.93±5.20
Bacteria	Brevundimonas	45.58±7.70	9.21±0.88	65.70±5.72
Bacteria	Nitratiruptor	45.32±13.31	0	117.17±5.92
Bacteria	Candidatus Puniceispirillum	45.14±9.77	12.56±0.46	49.15±4.08
Bacteria	Solibacillus	44.54±6.30	21.93±2.00	37.46±2.78
Bacteria	Thermovibrio	44.43±13.05	7.06±0.44	70.54±5.62
Bacteria	F_Alteromonadaceae	43.96±5.15	16.99±0.83	34.09±3.04
Bacteria	Lacinutrix	43.28±9.84	5.61±0.76	50.64±1.62
Bacteria	Sulfurospirillum	42.78±12.24	0.07±0.07	39.20±3.07
Bacteria	Thermobifida	42.40±7.12	41.70±1.87	49.79±4.64
Bacteria	Oceanithermus	42.28±2.10	66.91±1.34	34.96±2.90
Bacteria	Ureaplasma	42.18±11.87	11.14±2.24	173.10±18.43
Bacteria	Candidatus Protochlamydia	41.64±2.29	7.73±0.54	27.59±0.50
Bacteria	O_Chlamydiales	41.15±4.58	16.74±2.66	18.53±1.03
Bacteria	Methylophaga	40.78±7.42	6.76±0.26	70.87±6.31
Bacteria	Roseiflexus	40.57±11.12	6.34±0.64	36.01±3.32
Bacteria	Thermodesulfatator	39.94±5.52	2.82±0.59	14.42±1.08
Bacteria	Lawsonia	38.80±6.99	27.30±2.62	97.63±6.90
Bacteria	Saccharophagus	38.72±2.54	14.79±0.75	46.48±3.33
Bacteria	Hydrogenobacter	38.59±10.33	6.58±0.10	13.04±1.18
Bacteria	Herminiimonas	38.26±6.47	3.13±0.42	41.74±3.52
Bacteria	Croceibacter	38.14±6.91	49.68±5.66	36.71±1.92
Bacteria	Thermovirga	38.13±9.52	6.88±0.35	48.01±3.59
Bacteria	F_Streptococcaceae	38.00±6.80	28.78±2.68	28.07±2.45
Bacteria	Waddlia	37.83±2.08	22.66±0.50	27.86±3.08
Bacteria	Ferrimonas	37.57±6.55	102.31±1.57	41.84±3.35
Bacteria	Kangiella	37.52±3.20	32.18±2.83	49.01±2.97
Bacteria	Isosphaera	37.37±8.80	0.07±0.03	31.53±2.83

Bacteria	Neorickettsia	37.32±7.64	28.16±1.93	13.77±1.09
Bacteria	Calditerrivibrio	37.21±9.42	10.33±0.67	26.44±1.77
Bacteria	Gallibacterium	37.21±3.96	76.87±7.83	102.57±6.75
Bacteria	Haemophilus	36.78±5.46	22.13±1.95	79.49±8.46
Bacteria	Acetohalobium	36.60±6.64	18.69±1.77	34.43±2.29
Bacteria	Nitrobacter	36.51±3.49	143.92±7.55	20.26±1.90
Bacteria	Brucella	36.42±2.23	30.93±1.21	29.70±3.05
Bacteria	Acidothermus	36.01±4.34	15.41±0.44	27.18±2.56
Bacteria	Anaerolinea	35.54±4.09	16.32±1.53	51.29±3.27
Bacteria	Tolumonas	35.44±2.61	78.36±2.61	50.73±3.45
Bacteria	Azotobacter	35.23±2.15	71.75±3.13	35.45±2.08
Bacteria	F_Rhodobacteraceae	35.15±4.99	8.51±0.73	28.66±3.01
Bacteria	Candidatus Blochmannia	35.06±2.59	31.13±2.41	20.08±1.77
Bacteria	Photorhabdus	35.05±1.93	65.65±3.42	27.41±1.93
Bacteria	Thermoanaerobacter	34.93±1.78	14.80±2.05	35.97±1.35
Bacteria	Edwardsiella	34.43±2.76	22.96±0.70	31.81±2.89
Bacteria	F_Cytophagaceae	34.29±7.37	2.29±0.18	46.99±3.77
Bacteria	Asticcacaulis	34.22±7.53	9.67±1.49	39.77±4.19
Bacteria	Caldisericum	34.21±7.54	10.11±0.96	50.45±2.39
Bacteria	Micavibrio	33.99±8.34	4.30±0.37	39.92±4.80
Bacteria	Desulfurivibrio	33.87±6.12	15.13±1.20	19.30±2.56
Bacteria	F_Planctomycetaceae	33.60±9.63	0.29±0.20	46.14±3.75
Bacteria	Pantoea	33.55±8.17	27.67±0.47	54.57±4.62
Bacteria	Ilyobacter	33.41±7.99	11.78±1.19	36.24±2.04
Bacteria	Candidatus Sulcia	33.38±5.29	38.94±5.08	35.39±1.24
Bacteria	Xanthomonas	33.07±3.88	150.29±2.07	99.60±6.36
Bacteria	Thermodesulfovibrio	32.03±7.42	1.41±0.56	43.61±2.39
Bacteria	Candidatus Hodgkinia	31.99±7.08	0.15±0.03	30.21±2.01
Bacteria	Haliangium	31.32±5.09	26.94±1.26	17.69±2.40
Bacteria	F_Brucellaceae	31.16±4.73	15.86±0.64	28.34±2.43
Bacteria	Phycisphaera	30.71±7.86	1.17±0.14	12.05±1.60
Bacteria	Azoarcus	30.44±1.93	76.81±3.25	15.75±1.71
Bacteria	Thermaerobacter	30.31±1.21	7.27±0.49	29.59±3.68
Bacteria	C_Epsilonproteobacteria	30.28±8.62	0.12±0.05	35.78±2.64
Bacteria	Anaerococcus	30.13±5.05	4.29±0.18	52.16±1.02
Bacteria	Aquifex	29.99±8.20	178.90±15.27	35.87±3.30
Bacteria	Dehalogenimonas	29.98±8.42	0	20.81±1.84
Bacteria	Krokinobacter	29.65±2.42	20.23±1.13	26.58±1.72

Bacteria	Anabaena	29.49±8.19	0.03±0.02	31.39±2.01
Bacteria	Chromohalobacter	29.46±0.97	31.39±0.88	22.31±1.49
Bacteria	Thermomonospora	29.08±4.31	4.77±0.93	15.55±2.47
Bacteria	F_Mycoplasmataceae	28.33±8.55	0	104.44±10.15
Bacteria	Arcanobacterium	28.23±8.45	0	32.77±2.59
Bacteria	Oceanimonas	28.07±2.94	13.01±1.02	22.20±2.57
Bacteria	Meiothermus	27.85±7.48	1.99±0.33	44.73±2.98
Bacteria	Jannaschia	27.80±2.56	5.37±0.61	26.67±2.93
Bacteria	Cellvibrio	27.74±7.84	2.49±0.43	31.29±2.90
Bacteria	F_Pseudomonadaceae	27.60±0.87	88.38±5.22	27.47±2.92
Bacteria	O_Rhodobacterales	27.14±2.95	3.04±0.58	22.78±2.09
Bacteria	Frateuria	27.06±1.22	15.28±1.09	23.06±2.61
Bacteria	Sphaerobacter	27.02±2.56	52.21±3.70	18.91±1.69
Bacteria	Erwinia	26.98±1.75	10.51±0.74	14.07±1.61
Bacteria	Thermosediminibacter	26.84±1.33	40.08±1.63	20.93±1.83
Bacteria	Histophilus	26.64±2.03	6.24±0.81	29.26±1.82
Bacteria	Candidatus Accumulibacter	26.63±5.33	18.87±2.32	28.79±2.61
Bacteria	F_Halomonadaceae	25.82±4.01	9.15±0.49	24.13±2.32
Bacteria	O_Aquificales	25.75±7.67	0	23.46±1.36
Bacteria	Phaeobacter	25.47±0.98	17.65±1.28	26.74±2.70
Bacteria	Rhodopseudomonas	25.40±7.05	6.27±0.27	21.16±1.76
Bacteria	Ruegeria	25.28±0.85	12.75±0.38	30.83±2.38
Bacteria	Psychrobacter	24.90±6.94	0	37.96±3.63
Bacteria	Thermoanaerobacterales Family III	24.79±2.49	47.15±5.53	27.42±1.34
Bacteria	O_Campylobacterales	24.54±6.85	1.00±0.16	32.83±2.45
Bacteria	F_Vibrionaceae	24.52±5.94	0	20.73±1.26
Bacteria	Providencia	24.29±1.02	9.85±1.60	32.70±1.26
Bacteria	Weissella	24.22±6.89	3.09±0.33	1.45±0.17
Bacteria	Anaplasma	23.98±6.97	2.67±0.32	36.57±2.48
Bacteria	O_Rhodospirillales	23.60±5.41	7.77±0.68	13.92±1.67
Bacteria	Delftia	23.39±3.16	114.15±7.46	34.85±3.97
Bacteria	Oligotropha	22.99±3.31	6.35±0.85	13.57±0.63
Bacteria	F_Chroococcales	22.94±6.43	0.05±0.05	27.65±2.48
Bacteria	Thiomicrospira	22.77±6.60	0	11.91±1.19
Bacteria	Halorhodospira	22.24±2.57	21.50±1.27	14.49±1.47
Bacteria	F_Chroococcales	21.95±5.14	11.91±1.16	10.52±0.34
Bacteria	Wolbachia	21.76±1.68	45.55±3.70	11.34±0.48

Bacteria	Kocuria	21.40±6.45	0	7.45±0.48
Bacteria	Persephonella	20.52±4.22	3.07±0.45	36.09±2.38
Bacteria	Magnetococcus	20.46±4.80	1.00±0.23	26.17±2.64
Bacteria	Truepera	20.38±6.07	0	22.74±1.18
Bacteria	Cronobacter	20.13±3.45	15.57±0.65	13.58±1.65
Bacteria	Gloeobacter	19.81±2.08	7.90±0.19	48.93±3.49
Bacteria	F_Staphylococcaceae	19.22±4.72	1.92±0.27	14.98±1.55
Bacteria	Acidiphilium	19.04±0.93	6.18±0.20	7.94±0.75
Bacteria	O_Sphingobacteriales	18.96±4.73	5.12±0.35	25.17±1.47
Bacteria	Hippea	18.70±4.10	13.24±0.95	18.86±1.19
Bacteria	Anoxybacillus	18.69±5.70	0	29.70±2.55
Bacteria	F_Thermoanaerobacteraceae	18.62±2.72	8.40±0.65	20.38±1.41
Bacteria	F_Chlamydiaceae	18.38±5.52	9.09±0.24	15.68±2.05
Bacteria	F_Oxalobacteraceae	18.26±5.18	0.74±0.23	14.27±1.18
Bacteria	Proteus	18.23±2.96	13.83±1.57	13.89±0.89
Bacteria	Candidatus Riesia	18.01±4.04	1.27±0.18	8.84±0.79
Bacteria	F_Microbacteriaceae	17.99±2.66	18.34±1.06	15.20±0.92
Bacteria	F_Enterococcaceae	17.81±4.78	0.07±0.07	8.82±0.38
Bacteria	Dichelobacter	17.26±3.58	1.55±0.36	40.03±5.62
Bacteria	F_Helicobacteraceae	17.22±5.23	0	5.20±0.47
Bacteria	Halothermothrix	17.06±5.36	0.71±0.10	20.15±1.06
Bacteria	Amphibacillus	16.59±4.41	11.96±1.20	15.95±0.70
Bacteria	Thermomicrobium	16.35±4.55	3.21±0.32	10.65±1.25
Bacteria	Gramella	16.30±1.98	19.61±2.50	16.10±1.40
Bacteria	F_Acetobacteraceae	16.28±0.54	16.10±0.74	9.95±0.94
Bacteria	F_Parachlamydiaceae	16.02±5.57	1.31±0.39	5.52±0.30
Bacteria	Herpetosiphon	15.09±4.44	0	31.68±3.00
Bacteria	Taylorella	14.99±1.63	0.06±0.04	50.84±4.94
Bacteria	F_Leptotrichiaceae	14.86±4.58	0	17.28±1.84
Bacteria	Sulfurihydrogenibium	14.74±3.67	1.46±0.15	37.47±1.77
Bacteria	F_Rhodocyclaceae	14.72±4.41	0	9.12±0.72
Bacteria	Sodalis	14.66±1.69	7.12±0.76	31.47±2.41
Bacteria	Collimonas	14.47±1.99	0.60±0.07	9.87±0.68
Bacteria	F_Cyclobacteriaceae	14.31±2.93	2.47±0.65	22.01±1.97
Bacteria	Idiomarina	14.27±0.83	15.91±0.58	15.36±1.79
Bacteria	F_Methylophilaceae	13.83±4.04	2.56±0.07	27.90±2.92
Bacteria	Microcystis	13.74±4.18	0	8.04±0.68
Bacteria	Pseudoalteromonas	13.41±2.26	25.64±0.76	27.36±1.67

Bacteria	Brachybacterium	13.24±1.39	3.65±0.60	9.57±1.06
Bacteria	Marinithermus	13.02±2.33	14.38±1.21	15.10±0.79
Bacteria	F_Ectothiorhodospiraceae	12.31±3.45	88.08±1.31	5.65±0.76
Bacteria	F_Aquificaceae	12.24±3.88	0	6.26±0.60
Bacteria	Carboxydothermus	11.94±2.62	15.24±1.78	16.95±1.13
Bacteria	Beutenbergia	11.93±0.64	10.99±1.00	6.55±0.63
Bacteria	Natranaerobius	11.84±2.24	2.09±0.41	12.63±0.63
Bacteria	Candidatus Cloacamonas	11.71±3.55	0	18.87±1.28
Bacteria	Psychromonas	11.55±2.10	20.47±1.85	12.81±0.99
Bacteria	Photobacterium	11.03±0.47	0.85±0.20	8.01±0.29
Bacteria	F_Sphingobacteriales	10.76±1.94	2.67±0.21	10.91±0.78
Bacteria	Microlunatus	10.34±1.25	1.27±0.21	15.53±1.21
Bacteria	Chlamydophila	10.23±2.74	0.80±0.15	8.20±0.68
Bacteria	Thermosynechococcus	10.21±2.77	6.90±0.52	19.25±1.85
Bacteria	C_Chloroflexi	10.08±2.99	0	8.57±1.11
Bacteria	Thiomonas	9.93±1.82	3.30±0.29	11.15±1.43
Bacteria	Pediococcus	9.74±0.66	1.87±0.04	7.74±0.79
Bacteria	C_Deinococci	9.17±2.72	0	16.28±1.72
Bacteria	Tetragenococcus	8.93±0.71	0.18±0.18	5.07±0.42
Bacteria	Flexistipes	8.71±1.41	0	6.97±0.86
Bacteria	Methylotenera	8.65±1.53	5.87±0.87	13.19±1.81
Bacteria	Polynucleobacter	8.63±0.53	32.83±1.22	11.74±1.20
Bacteria	Pasteurella	8.56±0.90	22.40±2.12	11.69±1.06
Bacteria	F_Veillonellaceae	8.51±1.87	2.78±0.38	16.04±1.63
Bacteria	F_Synergistaceae	8.24±2.44	3.51±0.39	15.28±1.04
Bacteria	F_Methylococcaceae	8.19±1.58	0.27±0.02	5.01±1.05
Bacteria	Laribacter	8.17±1.06	2.09±0.27	11.67±1.84
Bacteria	Aerococcus	8.09±1.28	0.82±0.09	7.02±0.38
Bacteria	Nocardia	8.01±2.47	0	6.19±0.52
Bacteria	F_Chloroflexaceae	7.99±2.43	0	8.17±0.72
Bacteria	Moraxella	7.81±2.34	0	39.37±4.30
Bacteria	P_Elusimicrobia	7.61±2.39	0	10.05±0.68
Bacteria	F_Lactobacillaceae	7.60±2.05	1.38±0.02	8.30±0.51
Bacteria	Bordetella	7.51±0.59	3.05±0.76	19.19±0.58
Bacteria	Citrobacter	7.36±0.57	3.25±0.99	4.64±0.32
Bacteria	F_Alcaligenaceae	7.27±0.79	1.36±0.18	10.63±0.79
Bacteria	Acetobacter	7.26±2.16	0	7.70±1.15
Bacteria	Candidatus Portiera	7.25±1.93	2.11±0.71	8.29±0.36

Bacteria	F_Gallionellaceae	7.17±2.15	0	2.93±0.22
Bacteria	Stenotrophomonas	7.09±2.01	3.18±0.70	12.70±1.50
Bacteria	Thermodesulfobium	7.07±0.64	25.01±2.58	13.78±1.06
Bacteria	O_Oceanospirillales	7.03±1.22	6.70±0.31	12.50±1.19
Bacteria	F_Anaplasmataceae	6.61±1.32	2.80±0.22	6.99±1.28
Bacteria	F_Moraxellaceae	6.60±2.00	0	6.12±0.93
Bacteria	Candidatus Pelagibacter	6.59±2.00	0	4.78±0.31
Bacteria	Saprospira	6.57±1.95	0	3.97±0.36
Bacteria	Simiduia	6.57±1.32	5.13±0.51	11.48±0.71
Bacteria	Acidimicrobium	6.56±1.06	8.66±1.23	3.94±0.39
Bacteria	Micrococcus	6.52±1.94	0	10.80±0.67
Bacteria	F_Sphingomonadaceae	6.45±1.50	1.23±0.27	6.28±0.52
Bacteria	Synechocystis	6.37±1.91	0	4.52±0.35
Bacteria	Desulfarculus	6.36±1.84	0.93±0.27	7.10±1.24
Bacteria	Maricaulis	6.08±1.80	0	8.64±0.69
Bacteria	Renibacterium	6.03±1.80	0	4.04±0.53
Bacteria	F_Micrococcaceae	6.00±1.87	0	11.73±0.64
Bacteria	Salinispora	5.99±1.55	26.94±0.93	8.60±0.97
Bacteria	Pelagibacterium	5.97±1.78	0	25.70±0.65
Bacteria	F_Nocardiaceae	5.66±1.43	0.20±0.14	5.81±0.58
Bacteria	Cellulomonas	5.58±1.73	0	2.58±0.33
Bacteria	Tropheryma	5.58±1.70	0	1.90±0.33
Bacteria	Gordonia	5.38±0.44	3.97±0.88	4.26±0.66
Bacteria	Nocardioides	5.25±0.49	4.08±0.32	4.52±0.23
Bacteria	F_Mycobacteriaceae	5.17±1.07	1.14±0.14	5.74±0.49
Bacteria	Candidatus Hamiltonella	5.17±0.75	0	4.19±0.42
Bacteria	Sphingopyxis	5.17±1.22	0.87±0.19	8.81±1.15
Bacteria	Kineococcus	4.97±1.61	0	1.17±0.20
Bacteria	Kytococcus	4.80±1.10	0.90±0.37	5.16±0.75
Bacteria	Syntrophomonas	4.65±0.12	5.75±0.77	8.03±0.91
Bacteria	Frankia	4.39±1.06	0	11.68±1.12
Bacteria	Tsukamurella	4.26±1.24	1.61±0.08	3.41±0.28
Bacteria	F_Nitrosomonadaceae	4.07±0.72	0.15±0.10	0.94±0.13
Bacteria	F_Epsilonproteobacteria	4.05±1.29	0	1.06±0.16
Bacteria	Sphingomonas	3.93±1.16	0	2.78±0.39
Bacteria	F_Streptomycetaceae	3.89±1.16	0	3.62±0.46
Bacteria	Tistrella	3.80±0.53	0.77±0.31	5.07±0.76
Bacteria	Sanguibacter	3.70±0.19	26.36±2.61	3.69±0.50

Bacteria	F_Pseudonocardiaceae	3.67±0.87	0.09±0.05	1.30±0.24
Bacteria	F_Thermaceae	3.64±1.08	0	2.08±0.32
Bacteria	Allochromatium	3.61±1.12	0	9.95±1.35
Bacteria	F_Enterobacteriaceae	3.59±1.08	0.80±0.01	3.71±0.59
Bacteria	P_Alphaproteobacteria	3.47±1.05	0	5.02±0.35
Bacteria	Isoptericola	3.45±0.87	13.33±1.09	2.77±0.24
Bacteria	O_Halanaerobiales	3.42±1.11	0	5.88±0.75
Bacteria	F_Deferribacteraceae	3.38±1.03	0	2.53±0.28
Bacteria	Mesoplasma	3.28±0.23	10.97±1.06	4.61±0.33
Bacteria	Thiocystis	2.99±0.95	0	3.45±0.24
Bacteria	Saccharopolyspora	2.86±0.88	6.66±0.22	4.35±0.75
Bacteria	F_Campylobacteraceae	2.71±0.90	0	6.66±0.71
Bacteria	Actinosynnema	2.67±0.48	0	1.14±0.29
Bacteria	Candidatus Moranella	2.57±0.78	2.84±0.50	4.18±0.39
Bacteria	Actinoplanes	2.54±0.79	0	3.49±0.13
Bacteria	Cycloclasticus	2.52±0.31	0	5.82±0.51
Bacteria	Trichormus	2.48±0.78	1.14±0.36	4.10±0.38
Bacteria	P_Chloroflexi	2.35±0.74	0	3.54±0.22
Bacteria	O_Rickettsiales	2.13±0.44	0.12±0.02	4.39±0.40
Bacteria	Paracoccus	1.97±0.35	0	6.58±0.37
Bacteria	Pseudoxanthomonas	1.90±0.48	0.94±0.44	1.12±0.17
Bacteria	F_Lachnospiraceae	1.81±0.25	1.12±0.14	1.80±0.35
Bacteria	Hydrogenobaculum	1.79±0.57	0	5.42±0.51
Bacteria	F_Aeromonadaceae	1.79±0.56	0	8.58±0.65
Bacteria	Candidatus Amoebophilus	1.76±0.57	0	1.15±0.36
Bacteria	Nakamurella	1.66±0.54	0	2.99±0.37
Bacteria	F_Micromonosporaceae	1.57±0.47	0.03±0.03	0
Bacteria	F_Rhodothermaceae	1.44±0.46	0	1.05±0.18
Bacteria	Rubrivivax	1.39±0.46	0	1.86±0.43
Bacteria	Kribbella	1.39±0.45	0	1.38±0.12
Bacteria	Xylanimonas	1.35±0.36	13.67±0.87	0.18±0.06
Bacteria	F_Propionibacteriaceae	1.35±0.43	0	2.16±0.34
Bacteria	F_Beijerinckiaceae	1.34±0.45	0	2.13±0.22
Bacteria	O_Burkholderiales	1.34±0.41	0	2.05±0.21
Bacteria	Erythrobacter	1.15±0.37	0	1.04±0.22
Bacteria	Thioalkalimicrobium	1.11±0.37	0	0.71±0.26
Bacteria	Ketogulonicigenium	1.01±0.34	0	9.63±0.43
Bacteria	Verminephrobacter	0.95±0.12	0.36±0.16	0.87±0.17

Bacteria	F_Xanthomonadaceae	0.94±0.34	0	1.76±0.15
Bacteria	O_Sphingomonadales	0.91±0.33	0	0.66±0.14
Bacteria	F_Nocardioidaceae	0.85±0.29	0	1.61±0.15
Bacteria	F_Cryomorphaceae	0.85±0.28	0	2.45±0.22
Bacteria	C_Thermomicrobia	0.79±0.24	0	0.42±0.12
Bacteria	Verrucosispora	0.65±0.20	0	0
Bacteria	F_Hyphomonadaceae	0.59±0.23	0	0.59±0.20
Bacteria	Leptothrix	0.57±0.19	0	3.00±0.48
Bacteria	Clostridiales Family_XI	0.57±0.21	0	0.22±0.06
Bacteria	F_Piscirickettsiaceae	0.55±0.18	0	0.63±0.13
Bacteria	F_Ignavibacteriaceae	0.49±0.18	0	0.20±0.12
Bacteria	P_Acidobacteria	0.48±0.16	0	0.20±0.06
Bacteria	Candidatus Midichloria	0.42±0.15	0	0.05±0.02
Bacteria	F_Promicromonosporaceae	0.42±0.14	0	0.07±0.03
Bacteria	F_Hydrogenothermaceae	0.41±0.14	0	2.04±0.36
Bacteria	O_Deinococcales	0.38±0.13	0	1.31±0.24
Bacteria	F_Geodermatophilaceae	0.33±0.13	0	0.06±0.06
Bacteria	Kitasatospora	0.33±0.12	0	0.59±0.14
Bacteria	F_Saprospiraceae	0.32±0.11	0	0.25±0.05
Bacteria	F_Alicyclobacillaceae	0.31±0.14	0	0.84±0.12
Bacteria	F_Neisseriaceae	0.29±0.11	0	0.07±0.05
Bacteria	F_Halanaerobiaceae	0.20±0.09	0	0.14±0.09
Bacteria	F_Leuconostocaceae	0.11±0.06	0	0.83±0.05
Bacteria	Shigella	0.10±0.06	0	4.31±0.28
Bacteria	F_Nocardiopsaceae	0.05±0.04	0	0.92±0.21
Bacteria	P_Cyanobacteria	0.04±0.04	0	0.06±0.06
Bacteria	P_Gammaproteobacteria	0.04±0.03	0	0.22±0.07
Bacteria	F_Desulfobulbaceae	0.03±0.02	0	0.17±0.02
Bacteria	Thermobispora	0.03±0.02	0	0.22±0.06

[1] mean value ± standard error; the relative abundance of genera was normalized to 1,000,000 for each sample.

F = family; O = order; C = class; P = phylum.

Supplementary Table 3. The completeness, contamination, strain heterogeneity and genome size of metagenome bins found in this study

Bin	Taxonomy	Completeness (%)	Contamination (%)	Strain heterogeneity (%)	Genome size (bp)
1	o_Clostridiales	95.97	0.67	0	2124973
2	f_Lachnospiraceae	93.55	6.48	17.65	3604597
3	o_Bacteroidales	90.98	7.97	20	2506697
4	k_Bacteria	93.17	14.54	0	4219428
5	k_Bacteria	86.84	14.4	37.5	3563432
6	k_Bacteria	85.33	16.62	35.21	3320092
7	o_Clostridiales	83.87	11.66	19.23	3265029
8	o_Clostridiales	81.6	13.34	70	4434229
9	o_Clostridiales	79.27	1.69	20	9052789
10	o_Clostridiales	78.86	3.36	16.67	3656693
11	o_Clostridiales	78.25	5.92	10	4889815
12	o_Clostridiales	77.78	0.67	0	4925429
13	k_Bacteria	76.91	6.25	33.33	4815116
14	f_Mycoplasmataceae	73.46	3.21	0	12684799
15	o_Selenomonadales	72.73	7.62	34.78	5706848
16	f_Lachnospiraceae	72.62	14.31	10	4510896
17	k_Bacteria	72.58	17.61	0	6118940
18	k_Bacteria	72.56	4.28	75	4330909
19	k_Bacteria	72.33	6.13	36.36	3350024
20	c_Clostridia	71.93	4.58	0	7830036
21	o_Clostridiales	70.83	2.75	42.11	7832690
22	c_Deltaproteobacteria	70.53	3.02	14.29	8317536
23	k_Bacteria	100	221.52	11.25	6269479
24	k_Bacteria	100	243.75	1.99	6713701
25	k_Bacteria	100	247.51	3.54	3906535
26	k_Bacteria	100	270.76	6.5	12651718
27	k_Bacteria	100	304.08	4.03	4788861
28	k_Bacteria	100	322.49	7.61	14913618
29	k_Bacteria	100	339.29	8.72	10084005
30	k_Bacteria	100	344.67	7.09	8227111
31	k_Bacteria	99.84	270.36	14.98	9042075
32	k_Bacteria	99.15	209	0.74	13759356
33	k_Bacteria	99.06	273.56	21.94	9420351
34	k_Bacteria	98.28	116.69	14.67	10968148
35	k_Bacteria	98.28	481.68	32.71	19225062
36	k_Bacteria	98.25	623.82	1.37	18623372
37	k_Bacteria	97.96	372.87	4.75	14481240
38	k_Bacteria	97.56	53.91	69.41	7293506
39	k_Bacteria	97.49	428.45	31.07	9349720

40	k_Bacteria	97.41	327.38	1.54	8370202
41	k_Bacteria	97.34	173.7	35.35	6935511
42	k_Bacteria	96.55	85.74	54.84	6486206
43	k_Bacteria	96.55	114.97	34.13	4910411
44	k_Bacteria	96.55	116.47	77.08	17596109
45	k_Bacteria	96.55	119.7	7.38	11361833
46	k_Bacteria	96.55	139.26	5.2	16071748
47	k_Bacteria	96.55	167.66	2.5	9316117
48	k_Bacteria	96.55	168.29	6.2	17582974
49	k_Bacteria	95.69	82.88	4.2	12823129
50	k_Bacteria	95.5	44.74	2	22917110
51	k_Bacteria	95.39	323.55	1.58	14952134
52	k_Bacteria	95.34	94.98	39.24	12755558
53	k_Bacteria	95.11	67.72	61.17	14309934
54	o_Bacteroidales	95.11	138.86	2.23	16192142
55	k_Bacteria	94.74	144.94	22.78	30217467
56	k_Bacteria	94.69	250.84	3.97	22257258
57	k_Bacteria	94.59	194.46	6.29	22181491
58	k_Bacteria	94.58	179.71	1.87	10690789
59	k_Bacteria	94.47	154.62	2.38	30615561
60	k_Bacteria	94.26	158.18	19.38	3863443
61	k_Bacteria	94.2	160.06	52.52	9559136
62	k_Bacteria	93.97	76.97	23.78	2699119
63	k_Bacteria	93.94	113.04	16.54	7264869
64	p_Bacteroidetes	93.5	160.94	19.51	5682068
65	k_Bacteria	93.1	71.47	3.03	8294333
66	k_Bacteria	93.1	74.71	53.06	4948937
67	k_Bacteria	92.79	101.77	17.65	6960513
68	k_Bacteria	92.63	143.87	13.23	7218051
69	k_Bacteria	92.55	60.06	1.75	6843690
70	k_Bacteria	92.46	68.85	6.94	3117731
71	k_Bacteria	91.38	61.82	3.49	2158197
72	k_Bacteria	91.23	122.06	15.48	2857745
73	k_Bacteria	91.23	139.84	6.32	6253843
74	k_Bacteria	90.57	88.51	2.96	4398125
75	o_Bacteroidales	90.5	82.02	47.01	2396674
76	k_Bacteria	90.35	140.46	2.48	12279428
77	k_Bacteria	90.22	137.95	9.52	4688594
78	k_Bacteria	90.03	51.26	1.3	4227834
79	k_Bacteria	89.58	165.04	1.35	3996107
80	k_Bacteria	89.47	44.02	4.9	8899438
81	k_Bacteria	89.29	148.24	2.02	2337300
82	k_Bacteria	88.79	106.93	11.55	2766675

83	k_Bacteria	88.79	109.64	32.05	2668641
84	k_Bacteria	88.79	201.18	64.12	2410580
85	k_Bacteria	87.55	147.1	76.12	2785419
86	k_Archaea	87.54	127.08	41.83	7400807
87	k_Bacteria	87.43	93.58	7.19	5008816
88	k_Bacteria	86.62	63.57	5.71	5715702
89	k_Bacteria	86.41	110.88	15.21	9878788
90	k_Bacteria	86.36	128.29	32.92	1540738
91	k_Bacteria	86.21	76.72	4.4	4122962
92	k_Bacteria	86.05	145.76	4.86	25547918
93	k_Bacteria	85.66	62.76	50	6605188
94	k_Bacteria	85.5	76.47	11.24	18304670
95	k_Bacteria	84.98	94.38	2.17	11322465
96	k_Bacteria	84.76	66.25	1.09	6708682
97	p_Bacteroidetes	84.71	114.33	33.21	3286127
98	k_Bacteria	84.66	37.1	30.77	2008389
99	o_Clostridiales	84.54	30.77	2.53	3104707
100	k_Bacteria	84.48	170.89	22.61	5016236
101	o_Bacteroidales	83.76	99.24	8.78	2170116
102	k_Bacteria	83.46	68.97	67.42	1290699
103	k_Bacteria	82.55	108.17	1.49	5976822
104	k_Bacteria	82.41	91.96	6.93	4250155
105	k_Bacteria	82.29	76.4	15.91	1972962
106	k_Bacteria	81.97	140.67	7.18	5993065
107	k_Bacteria	81.47	42.81	3.66	2176560
108	k_Bacteria	81.39	30.17	0	12780205
109	k_Bacteria	80.97	24.87	52.17	1428914
110	k_Bacteria	80.78	89.81	8.12	1902776
111	k_Bacteria	80.72	128.26	19.42	7594736
112	k_Bacteria	80.06	73.73	4.31	5169434
113	o_Bacteroidales	78.87	36.87	8.57	7868718
114	k_Bacteria	77.59	73.45	23.31	11678726
115	k_Bacteria	77.59	108.66	14.78	2446172
116	o_Clostridiales	77.41	37.61	0	987771
117	k_Bacteria	77.25	75.25	3.04	7469443
118	k_Bacteria	77.02	69.75	24.32	2572645
119	f_Lachnospiraceae	76.64	29.02	0	2832435
120	k_Bacteria	76.12	96.79	1.24	1946513
121	k_Bacteria	74.84	139.81	36.53	3793676
122	k_Bacteria	73.43	49.92	25.53	6227729
123	o_Clostridiales	72.45	27.24	50	2319502
124	k_Bacteria	71.21	65.17	26.18	5160651
125	c_Clostridia	71	43.98	31	1787981
126	k_Bacteria	70.64	56.06	1.01	4911024

127	k_Bacteria	68.97	58.22	15.98	1470985
128	k_Bacteria	67.95	67.42	52.62	4545922
129	p_Bacteroidetes	67.51	26.97	9.28	2503056
130	o_Clostridiales	67.11	21.25	12.28	2130919
131	o_Clostridiales	66.8	9.21	77.78	2436136
132	k_Bacteria	66.61	32.68	0	3428419
133	k_Bacteria	65.82	29.78	42.37	2266656
134	p_Bacteroidetes	64.98	14.84	30.77	2156195
135	k_Bacteria	64	44.34	2.11	2235749
136	o_Clostridiales	63.84	34.26	0.99	2204966
137	k_Bacteria	63.64	49.12	11.65	4933765
138	f_Spirochaetaceae	63.49	0.55	0	3555272
139	k_Bacteria	62.62	17.02	7.69	1338405
140	o_Selenomonadales	61.96	2.4	20	2133376
141	o_Clostridiales	60.75	27.74	1.12	22775034
142	f_Lachnospiraceae	60.11	2.94	0	1429000
143	o_Clostridiales	59.74	5.94	0	1990587
144	o_Bacteroidales	59.01	8.83	11.63	3185420
145	k_Bacteria	58.78	61.9	40.64	820202
146	p_Firmicutes	58.56	1.32	33.33	1418416
147	o_Clostridiales	58.4	14.04	6.82	2247063
148	k_Bacteria	58.24	14.66	22.22	1503166
149	k_Bacteria	57.02	79.88	9.11	2556519
150	k_Bacteria	56.33	20.91	17.07	1381725
151	o_Bacteroidales	56.2	4.47	11.11	1438346
152	o_Bacteroidales	50.22	2.88	58.33	1870290
153	o_Bacteroidales	50.17	0.94	66.67	2653203
154	k_Bacteria	48.75	10.34	16.67	2816454
155	o_Clostridiales	47.39	13.79	5.08	1168869
156	o_Clostridiales	46.13	13.46	0	1582824
157	k_Bacteria	45.61	7.15	9.52	1721137
158	p_Bacteroidetes	44.76	2.32	0	2417916
159	c_Clostridia	43.85	10.35	16.67	6849613
160	k_Bacteria	43.34	13.09	6.25	2650926
161	k_Bacteria	42.99	23.98	8.11	922016
162	o_Clostridiales	42.07	4.5	10	1384945
163	o_Clostridiales	42.06	5.78	42.86	4101605
164	k_Bacteria	40.87	9.4	81.48	1330426
165	k_Bacteria	37.8	8.93	61.4	1337168
166	k_Bacteria	37.42	2.51	0	561039
167	o_Clostridiales	37.08	4.86	33.33	1021049
168	k_Bacteria	36.12	5.26	50	974998
169	k_Bacteria	35.63	3.48	0	659850

170	k_Bacteria	35.58	4.31	33.33	1247812
171	k_Bacteria	35.11	1.72	0	2343479
172	k_Bacteria	34.96	1.91	0	1061788
173	k_Bacteria	34.09	13.95	0	1360934
174	k_Bacteria	31.98	0.79	0	879079
175	k_Bacteria	31.67	7.02	83.33	1403938
176	o_Clostridiales	30.12	0.95	0	1703931
177	k_Bacteria	29.82	0	0	600905
178	k_Bacteria	28.21	0	0	4857729
179	k_Bacteria	27.86	18.71	10	1092701
180	k_Bacteria	24.89	14.56	44.44	1732802
181	k_Bacteria	24.2	4.61	16.67	834283
182	k_Bacteria	21.45	1.75	0	666465
183	k_Bacteria	19.44	0	0	4362654
184	f_Veillonellaceae	17.9	0.93	100	470524
185	k_Bacteria	17.85	0	0	651678
186	k_Bacteria	14.86	1.75	0	278267
187	k_Bacteria	13.73	4.55	37.04	1065255
188	k_Bacteria	12.82	5.81	15.79	786194
189	k_Bacteria	12.07	0	0	1747207
190	k_Bacteria	11.21	0	0	270519
191	k_Bacteria	9.48	8.78	20	653295
192	k_Bacteria	9.35	2.04	25	325556
193	k_Bacteria	8.26	0	0	1079397
194	k_Bacteria	6.82	0.31	0	276028
195	k_Bacteria	6.11	0	0	244880
196	k_Bacteria	5	1.72	0	564816
197	k_Bacteria	4.62	0.31	0	602622
198	k_Bacteria	3.43	0	0	1092918
199	k_Bacteria	3.11	0	0	403770
200	k_Bacteria	3.06	0	0	1048148
201	k_Bacteria	2.35	0	0	114517
202	k_Bacteria	1.96	3.45	11.84	988378
203	k_Bacteria	1.79	0	0	312262
204	k_Bacteria	1.72	0	0	2702150
205	k_Bacteria	0.32	0	0	71031
206	k_Bacteria	0.31	0	0	851454
207	k_Bacteria	0.16	0	0	1439485
208	k_Archaea	0.07	0	0	700788

k = kindom, p = phylum, 0 = order, c = class, f = family

Supplementary Table 4. Comparisons of the relative abundance of major contributors to the ETC components between groups.

ETCs	Major contributers	B[1]	G[1]	P[1]	G/B[2]	P/B[2]
ATPase	Prevotella	557.22±42.17	1053.57±75.77	980.00±3.13	0.92	0.81
ATPase	Geobacter	183.52±13.52	212.61±17.30	162.19±3.23	0.21	-0.18
ATPase	Slackia	25.56±3.24	166.61±13.46	66.79±3.71	2.70	1.39
ATPase	Paenibacillus	77.33±9.53	159.70±16.15	56.47±11.48	1.05	-0.45
ATPase	Sinorhizobium	16.87±1.30	46.10±9.76		1.45	
ATPase	Bacillus	69.41±10.66	41.85±2.70		-0.73	
ATPase	Desulfovibrio	34.06±2.79	41.21±7.46		0.27	
ATPase	Eggerthella	56.92±4.78	39.39±5.41	29.90±3.19	-0.53	-0.93
ATPase	Clostridium	252.85±23.55	33.81±1.23	60.53±2.48	-2.90	-2.06
ATPase	Syntrophobacter	43.03±4.06	25.43±1.82	34.99±6.55	-0.76	-0.30
ATPase	Arthrobacter	74.18±5.18	14.38±0.85	19.96±3.88	-2.37	-1.89
ATPase	Bacteroides	20.45±2.49		151.50±16.07		2.89
ATPase	Desulfomicrobium	6.68±0.84		28.66±5.79		2.10
ATPase	Selenomonas	6.47±1.07		21.94±2.90		1.76
ATPase	Bifidobacterium	4.50±0.42		16.16±2.40		1.84
Cyd	Prevotella	241.24±18.57	921.78±83.71	398.77±51.69	1.93	0.73
Cyd	Slackia	2.84±0.16	372.18±47.02		7.03	
Cyd	Granulicella	43.49±3.24	73.72±7.81	68.53±8.72	0.76	0.66
Cyd	Acidaminococcus	88.38±9.07	65.46±5.59		-0.43	
Cyd	Geobacter	11.27±1.28	32.43±3.81	39.08±3.04	1.52	1.79
Cyd	Bifidobacterium	0.11±0.03	32.11±4.84		8.19	
Cyd	Desulfurispirillum	1.93±0.21	11.42±2.21		2.56	
Cyd	Marinobacter	46.39±3.22	10.94±1.60	13.65±1.96	-2.08	-1.77
Cyd	Eggerthella	9.44±0.74	9.56±0.70		0.02	
Cyd	Arthrobacter	26.35±2.53	1.69±0.44	56.58±4.87	-3.96	1.10
Cyd	Paenibacillus	43.72±4.09	1.25±0.18		-5.13	
Cyd	Desulfomicrobium	13.52±1.09	0.18±0.08		-6.23	
Cyd	Syntrophobacter	24.99±1.82	0.10±0.03	24.63±2.41	-7.97	-0.02
Cyd	Methylobacillus	0.69±0.07		63.39±4.24		6.52
Cyd	Bacteroides	22.13±1.96		18.01±1.38		-0.30
Cyd	Terriglobus	0.44±0.06		14.80±1.86		5.07
Cyd	Comamonas	5.50±0.70		1.76±0.16		-1.64
NrfA	Prevotella	148.48±14.64	220.31±18.16	295.17±35.99	0.57	0.99
NrfA	Desulfovibrio	25.23±1.25	101.24±14.98		2.00	

NrfA	Shewanella	10.84±1.05	75.13±0.51		2.79	
NrfA	Syntrophobacter	16.03±0.47	52.77±6.40		1.72	
NrfA	Granulicella	14.78±2.01	20.29±0.60	13.59±2.45	0.46	-0.12
NrfA	Paenibacillus	3.18±0.21	19.60±1.70		2.62	
NrfA	Pseudovibrio	4.61±0.68	17.79±0.66		1.95	
Nuo	Prevotella	867.60±60.59	2079.64±232.52	1287.74±150.42	1.26	0.57
Nuo	Paenibacillus	20.94±2.22	48.91±3.99		1.22	
Nuo	Clostridium	20.55±2.53	42.36±3.01	21.15±0.98	1.04	0.04
Nuo	Bacteroides	3.04±0.44	22.51±1.29		2.89	
Nuo	Geobacter	3.90±0.18	19.77±1.94		2.34	
Nuo	Eggerthella	6.73±0.71	14.43±2.08		1.10	
Nuo	Slackia	17.42±1.43	12.41±0.85	13.41±1.71	-0.49	-0.38
Nuo	Fibrobacter	54.02±4.92		37.74±3.06		-0.52
Nuo	Aggregatibacter	1.09±0.13		14.74±1.67		3.76
Rnf	Prevotella	493.35±45.61	1375.76±116.42	829.70±91.58	1.48	0.75
Rnf	Slackia	40.18±3.47	246.37±17.75	27.76±2.16	2.62	-0.53
Rnf	Granulicella	42.57±4.78	181.08±18.35	53.64±3.49	2.09	0.33
Rnf	Azospira	23.43±1.74	92.03±9.44		1.97	
Rnf	Arthrobacter	187.60±16.09	76.32±8.30	90.91±10.90	-1.30	-1.05
Rnf	Paenibacillus	46.14±3.19	55.38±4.15	20.74±1.71	0.26	-1.15
Rnf	Clostridium	50.27±5.74	54.88±0.96	45.52±2.86	0.13	-0.14
Rnf	Geobacter	152.12±13.56	51.81±5.06	126.90±11.04	-1.55	-0.26
Rnf	Eggerthella	90.21±9.55	32.86±3.51	28.21±2.14	-1.46	-1.68
Rnf	Syntrophus	2.86±0.08	21.27±1.45		2.89	
Rnf	Bacteroides	58.15±5.95	19.70±0.05	122.12±9.00	-1.56	1.07
Rnf	Sinorhizobium	30.02±2.17	13.30±0.85	55.47±4.43	-1.17	0.89
Rnf	Desulfovibrio	46.79±3.68	10.92±0.66	41.91±5.70	-2.10	-0.16
Rnf	Alcanivorax	9.80±1.55	9.60±2.81		-0.03	
Rnf	Selenomonas	6.38±0.65	9.57±0.39		0.58	
Rnf	Bacillus	29.77±2.46	8.13±0.77	25.45±1.44	-1.87	-0.23
Rnf	Bradyrhizobium	9.22±1.40	5.50±0.84		-0.75	
Rnf	Sphaerochaeta	19.33±0.90	3.79±0.33	21.72±2.95	-2.35	0.17
Rnf	Bifidobacterium	9.47±0.84		15.82±1.93		0.74
Rnf	Pelodictyon	7.96±0.73		12.94±1.30		0.70
Rnf	Sorangium	7.68±0.79		10.16±0.52		0.40
Sdh	Prevotella	368.51±30.84	618.17±44.02	486.22±45.75	0.75	0.40
Sdh	Slackia	21.60±1.98	175.19±12.72	13.53±1.25	3.02	-0.68
Sdh	Aeromonas	2.57±0.28	126.36±11.62		5.62	

Sdh	Geobacter	124.27±9.84	117.59±10.09	115.41±10.27	-0.08	-0.11
Sdh	Eggerthella	49.95±4.68	91.27±11.65	17.25±1.26	0.87	-1.53
Sdh	Pelobacter	1.39±0.19	47.08±4.71		5.08	
Sdh	Desulfovibrio	7.89±0.76	43.40±5.54	15.71±1.91	2.46	0.99
Sdh	Syntrophobacter	11.19±1.00	35.19±3.45		1.65	
Sdh	Agrobacterium	3.84±0.52	21.96±1.82		2.52	
Sdh	Olsenella	6.88±0.57	20.60±1.89		1.58	
Sdh	Selenomonas	26.05±2.06	16.78±0.99	11.70±1.20	-0.63	-1.15
Sdh	Clostridium	3.54±0.55	15.69±1.09		2.15	
Sdh	Candidatus Koribacter	3.34±0.47	13.74±0.95		2.04	
Sdh	Granulicella	47.09±5.58	12.22±1.25	25.01±2.30	-1.95	-0.91
Sdh	Desulfomicrobium	62.10±4.47	11.89±0.35	54.93±4.97	-2.38	-0.18
Sdh	Bacteroides	16.19±1.29	5.42±0.62	30.19±2.60	-1.58	0.90
Sdh	Methanolobus	5.29±0.32	3.51±0.28		-0.59	
Sdh	Bifidobacterium	13.72±0.31	3.02±0.57		-2.18	
Sdh	Thermococcus	11.40±1.30	1.88±0.76		-2.60	
Sdh	Butyrivibrio	10.42±1.34	0.59±0.12		-4.14	
Sdh	Methylobacillus	2.04±0.15		66.30±5.06		5.02
Sdh	Tannerella	20.52±2.72		47.49±2.33		1.21
Sdh	Desulfomonile	3.17±0.26		33.29±1.75		3.39
Sdh	Coraliomargarita	12.92±1.25		28.46±1.44		1.14
Sdh	Vibrio	5.74±0.40		27.68±2.66		2.27
Sdh	Paenibacillus	13.20±0.50		25.99±3.51		0.98
Sdh	Sinorhizobium	5.41±0.43		24.18±2.43		2.16
Sdh	Pseudovibrio	5.34±0.81		13.24±1.55		1.31
Sdh	Terriglobus	6.03±0.54		12.82±0.55		1.09
Sdh	Spirochaeta	3.28±0.19		11.35±0.38		1.79
Sdh	Akkermansia	3.14±0.51		10.98±1.27		1.81
Sdh	Treponema	4.68±0.51		10.79±0.48		1.21
Sdh	Syntrophus	5.12±0.42		10.47±0.85		1.03

[1] mean value ± standard error; the relative abundance of species results was normalized to 1,000,000 for each sample

[2] Values are calculated from $\log_2(G/B)$ or $\log_2(P/B)$.

Blue indicates the value is significantly decreased when the diet shifted; red indicates the value is significantly increased when the diet shifted
